# Fatty Acid-Activated Proton Transport by Bisaryl Anion Transporters Depolarises Mitochondria and Reduces the Viability of MDA-MB-231 Breast Cancer Cells

**DOI:** 10.3390/biom13081202

**Published:** 2023-07-31

**Authors:** Edward York, Daniel A. McNaughton, Meryem-Nur Duman, Philip A. Gale, Tristan Rawling

**Affiliations:** 1 School of Mathematical and Physical Sciences, Faculty of Science, University of Technology Sydney, Sydney, NSW 2007, Australia; edward.york@uts.edu.au (E.Y.);; 2 School of Chemistry, The University of Sydney, Sydney, NSW 2006, Australia; 3 The University of Sydney Nano Institute (SydneyNano), The University of Sydney, Sydney, NSW 2006, Australia

**Keywords:** mitochondria, uncouplers, anion transporter, proton transport, membrane, anticancer

## Abstract

In respiring mitochondria, the proton gradient across the inner mitochondrial membrane is used to drive ATP production. Mitochondrial uncouplers, which are typically weak acid protonophores, can disrupt this process to induce mitochondrial dysfunction and apoptosis in cancer cells. We have shown that bisaryl urea-based anion transporters can also mediate mitochondrial uncoupling through a novel fatty acid-activated proton transport mechanism, where the bisaryl urea promotes the transbilayer movement of deprotonated fatty acids and proton transport. In this paper, we investigated the impact of replacing the urea group with squaramide, amide and diurea anion binding motifs. Bisaryl squaramides were found to depolarise mitochondria and reduce MDA-MB-231 breast cancer cell viability to similar extents as their urea counterpart. Bisaryl amides and diureas were less active and required higher concentrations to produce these effects. For all scaffolds, the substitution of the bisaryl rings with lipophilic electron-withdrawing groups was required for activity. An investigation of the proton transport mechanism in vesicles showed that active compounds participate in fatty acid-activated proton transport, except for a squaramide analogue, which was sufficiently acidic to act as a classical protonophore and transport protons in the absence of free fatty acids.

## 1. Introduction

Targeting mitochondria is a promising approach to the development of new anticancer agents, as cancer cell mitochondria are structurally and functionally distinct from those in noncancerous cells, and because mitochondria play pivotal roles in cellular metabolism and cell survival [1,2]. Importantly, mitochondria use nutrients to synthesise ATP via oxidative phosphorylation (OXPHOS), where nutrient oxidation feeds electrons into the electron transport chain (ETC), a series of proteins embedded in the inner mitochondrial membrane (MIM). Electron flow through the ETC pumps protons from the mitochondrial matrix into the intermembrane space, generating a proton gradient across the MIM. The resulting membrane potential (Δ_ΨM_) is used to drive protons back into the matrix through ATP synthase, which converts ADP to ATP [3,4]. Nutrient oxidation is therefore coupled to ATP synthesis by the proton gradient across the MIM. Mitochondrial uncouplers are compounds that disrupt OXPHOS by inducing proton leakage across the MIM, creating a futile cycle of energy expenditure that diminishes the Δ_Ψm_ and ATP synthesis [5]. The most common uncouplers are lipophilic weak acids, such as 2,4-dinitrophenol (DNP), which transport protons across the MIM via a protonophoric cycle that involves the direct protonation and deprotonation of the acidic group. A growing body of evidence indicates that mitochondrial uncouplers can selectively kill cancer cells [6,7,8], which has focused attention on their potential use as new anticancer agents [2].

**Figure 1:**
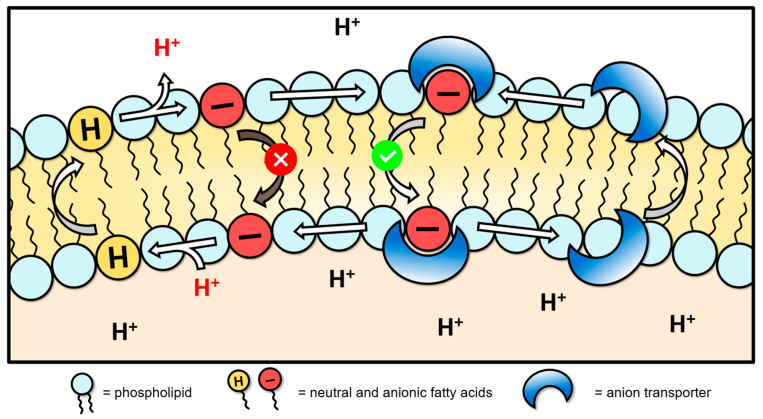
Fatty acid-activated proton transport mechanism via anion transporters in large unilamellar vesicles [9]. The anion transporter binds fatty acid carboxylates, masking their anionic charge to facilitate fatty acid flip-flop and proton transport across the membrane.

While most protonophores are weak acids, we have recently shown that synthetic anion transporters can also facilitate the movement of protons across lipid bilayers via a fatty acid-activated mechanism [9]. Anion transporters possess an anion binding motif that can form reversible intermolecular interactions with various anions, producing a lipophilic complex that facilities the passive transport of the anion across lipid bilayers [10]. Anion transporters promote transbilayer proton movement by interacting with free fatty acids within the membrane (see Figure 1) [9]. In the first step, the anionic form of the fatty acid accepts a proton from the relatively acidic solution and permeates the membrane as a neutral species. The fatty acid is deprotonated in the relatively alkaline solution, resulting in the transport of one proton and the regeneration of the membrane-impermeable carboxylate species. Next, the anion transporter binds to the carboxylate group through parallel hydrogen bonds to produce a lipophilic complex that is capable of diffusing through the bilayer. The disassociation of the complex is followed by the protonation of the fatty acid and further proton transport cycles.

The discovery of the fatty acid-activated proton transport mechanism was made using large unilamellar vesicles (LUVs), and we subsequently showed that the same mechanism can operate in the mitochondria of intact cells by utilising free fatty acids that are present in the MIM [11,12,13]. Thus, the treatment of MDA-MB-231 breast cancer cells with bisaryl urea-based synthetic anion transporters collapsed the proton gradient across the MIM and uncoupled OXPHOS, leading to a loss of cell viability [11]. The substitution of the bisaryl urea scaffold with lipophilic electron-withdrawing groups enhanced electrogenic proton transport using the fatty activated mechanism, as well as the mitochondrial and cellular effects [11]. This structure–activity relationship (SAR) aligns with the proposed transport mechanism as electron-withdrawing groups increase the urea N-H hydrogen bond donor strength and complexation with fatty acid anions, and lipophilic groups enhance the membrane permeability of the resulting complex.

**Figure 2:**
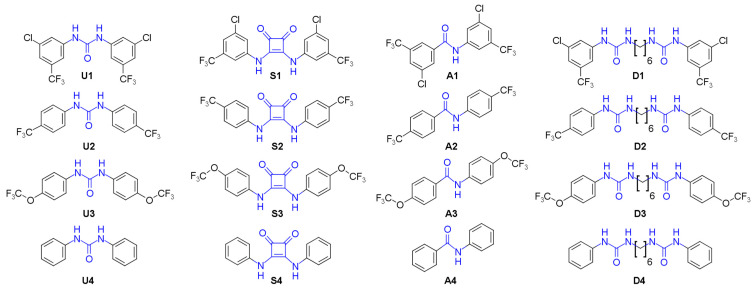
Chemical structures of bisaryl urea, squaramide, amide and diurea anion transporters studied in this work.

In this paper we replaced the anion binding motif in the bisaryl urea scaffold with squaramide [14,15], amide and diurea [16] groups (Figure 2) and studied the proton transport and mitochondrial uncoupling activities of these compounds. Bisaryl amides and diureas substituted with lipophilic electron-withdrawing groups showed enhanced proton transport by the fatty acid-activated mechanism, consistent with the established SAR. Bisaryl squaramides bearing these groups also show enhanced proton transport, but participated as both anion transporters and as classical protonophores, presumably due to the higher acidity of the squaramide motif. The analogues with the greatest proton transport activity uncoupled mitochondria, disrupting OXPHOS and reducing cell viability in MDA-MB-231 breast cancer cells.

## 2. Materials and Methods

### 2.1. Chemistry

**U1**–**4** were synthesised by the procedure in the literature [11]. 3-Chloro-5-(trifluoromethyl)benzoic acid, 4-(trifluoromethoxy)benzoic acid, 3,4-diethoxycyclobut-3-ene-1,2-dione and COMU were purchased from Fluorochem Limited (Hadfield, UK). The remaining reagents were purchased from Merck (Darmstadt, Germany). Reactions were monitored using TLC on Merck silica gel 60 F_254_ aluminium-backed plates. Reaction products were purified as required with dry column vacuum chromatography on silica gel using gradient elutions. NMR spectra were recorded using a Bruker 400 MHz and an Agilent 500 MHz NMR spectrometer, at operating frequencies of 400 and 500 MHz for ^1^H and 100 and 125 MHz for ^13^C spectra, respectively. Spectra were referenced internally to the residual solvent (CDCl_3_: ^1^H δ 7.26, ^13^C δ 77.16. DMSO-*d_6_*: ^1^H *δ* 2.50, ^13^C *δ* 39.52. CD_3_OD: ^1^H *δ* 3.31, ^13^C *δ* 49.00). Multiplicity was assigned as *s* (singlet), *d* (doublet), *t* (triplet), *q* (quartet), *qu* (quintet) and *m* (multiplet). High resolution mass spectra (HRMS) were recorded on an Agilent Technologies 6510 Q-TOF LCMS. The purity of all test compounds was confirmed to be >95% using absolute quantitative NMR spectroscopy (Supplementary Information, Table S1) [17].

#### 2.1.1. General Procedure for the Synthesis of Bisaryl Squaramides

To a solution of an appropriately substituted aniline (0.338 g, 2.1 mmol, 2.1 equiv.) in 2 mL 19:1 toluene:dimethyl formamide (DMF) was added the diethyl squarate ester (3,4-diethoxycyclobut-3-ene-1,2-dione, 0.170 g, 1.0 mmol, 1 equiv.) and zinc triflate (0.073 g, 0.2 mmol, 0.2 equiv.), and the resultant mixture was stirred for 18 h at 100 °C in a 10 mL sealed pressure tube. The desired squaramide products were found to precipitate out of the solution upon formation. The reaction mixture was pelleted via centrifugation and the supernatant was discarded, and then the crude solid was washed with 5 × 5 mL methanol using successive resuspension and centrifugation to give the desired products as white/yellow solids.

#### 2.1.2. General Procedure for the Synthesis of Bisaryl Amides

To a mixture of COMU ((1-cyano-2-ethoxy-2-oxoethylidenaminooxy)dimethylamino morpholino-carbenium hexafluorophosphate, 1.028 g, 2.4 mmol, 1.2 equiv.), triethylamine (0.405 g, 4.0 mmol, 2 equiv.) and an appropriately substituted benzoic acid (0.449 g, 2.0 mmol, 1.0 equiv.) in 5 mL DMF was added the corresponding substituted aniline (0.430 g, 2.2 mmol, 1.1 equiv.), and it was stirred for 24 h at room temperature under nitrogen. The resultant reaction mixtures were diluted ten-fold with water, extracted with 3 × 20 mL ethyl acetate and then washed with 3 × 20 mL of 1M hydrochloric acid followed by 3 × 20 mL saturated sodium bicarbonate. Crude solids were isolated with rotary evaporation and then purified via dry column vacuum chromatography (DCVC) using a gradient elution of Hexane/EtOAc (100:0 to 70:30).

#### 2.1.3. General Procedure for the Synthesis of Bisaryl Diureas

*N,N*′-Carbonyldiimidazole (1.14 g, 7 mmol, 1.4 equiv.) was added to a solution of an appropriately substituted aniline (0.466 g, 5 mmol, 1.0 equiv.) in anhydrous 1,2-dichloroethane (EDC, 7 mL) under N_2_. After stirring for 18 h, the resulting suspension was cooled on ice for 1 h, then filtered and washed with 1,2-dichloroethane to give the *N*-carbamoyl imidazole intermediate as a white solid. Next, the *N*-carbamoyl imidazole (0.562 g, 3.0 mmol, 3 equiv.) was resuspended in dichloromethane, hexamethylene diamine (0.116 g, 1 mmol, 1 equiv.) was added and it was stirred at room temperature for 18 h. The resultant suspension was filtered, and the solid obtained was washed with 10 mL dichloromethane. Residual aniline and the bisaryl urea byproduct were removed as required by trituration with 2 × 10 mL ethyl acetate as required.

*Bis({[3-chloro-5-(trifluoromethyl)phenyl]amino})cyclobut-3-ene-1,2-dione* (**S1**). ^1^H NMR (500 MHz, CD_3_OD) δ 7.78 (s, 2H), 7.71 (s, 2H), 7.37 (s, 2H). ^13^C NMR (125 MHz, CD_3_OD) δ 184.2 (2C), 167.2 (2C), 142.2 (2C), 137.1 (2C), 134.1 (q, *J* = 33 Hz, 2C), 124.5 (q, *J* = 271 Hz, 2C), 123.2 (2C), 121.0 (2C), 115.1 (q, *J* = 4 Hz, 2C). HRMS (ESI): *m*/*z* [M + H]^+^ calculated for C_18_H_8_Cl_2_F_6_N_2_O_2_: 468.9940, found: 468.9937.

*Bis({[4-(trifluoromethyl)phenyl]amino})cyclobut-3-ene-1,2-dione* (**S2**). 1H and 13C NMR and HRMS are in agreement with previously reported data [14,18].

*Bis({[4-(trifluoromethoxy)phenyl]amino})cyclobut-3-ene-1,2-dione* (**S3**). ^1^H NMR (500 MHz, DMSO-*d6*) δ 10.5 (br s, 2H), 7.59 (d, *J* = 8.5 Hz, 4H), 7.35 (d, *J* = 8 Hz, 4H) ^13^C NMR (125 MHz, DMSO-*d6*) δ 181.7 (2C), 165.7 (2C), 143.8 (2C), 137.9 (2C), 122.2 (4C), 120.1 (q, *J* = 255 Hz, 2H), 120.0 (4C). HRMS (ESI): *m*/*z* [M + H]^+^ calculated for C_18_H_10_F_6_N_2_O_4_: 433.0618, found: 433.0623.

*Bis(phenylamino)cyclobut-3-ene-1,2-dione* (**S4**). 1H and 13C NMR are in agreement with previously reported data [14]. HRMS (ESI): *m*/*z* [M + H]^+^ calculated for C_16_H_12_N_2_O_2_: 265.0972, found: 265.0958.

*3-Chloro-N-[3-chloro-5-(trifluoromethyl)phenyl]-5-(trifluoromethyl)benzamide* (**A1**). ^1^H NMR (500 MHz, CDCl_3_) δ 8.10 (br s, 1H), 8.04 (s, 1H), 7.99 (s, 1H), 7.98 (s, 1H), 7.82 (s, 1H), 7.77 (s, 1H), 7.43 (s, 1H). ^13^C NMR (125 MHz, CDCl_3_) δ 163.1 (1C), 138.8 (1C), 136.5 (1C), 136.1 (1C), 135.8 (1C), 133.1 (q, *J* = 34 Hz, 1C), 132.8 (q, *J* = 33 Hz, 1C), 130.8 (1C), 129.3 (q, *J* = 4 Hz, 1C), 123.47 (1C), 123.46 (1C), 122.9 (q, *J* = 271 Hz, 1C), 122.7 (q, *J* = 272 Hz, 1C), 122.1 (m, 2C), 115.3 (q, *J* = 4 Hz, 1C). HRMS (ESI): *m*/*z* [M + H]^+^ calculated for C_15_H_7_Cl_2_F_6_NO: 401.9882 found: 401.9883.

*4-(Trifluoromethyl)-N-[4-(trifluoromethyl)phenyl]benzamide* (**A2**). ^1^H and ^13^C NMR and HRMS are in agreement with previously reported data [19].

*4-(Trifluoromethoxy)-N-[4-(trifluoromethoxy)phenyl]benzamide* (**A3**). ^1^H NMR (500 MHz, DMSO-*d6*) δ 10.53 (s, 1H), 8.08 (AA’BB’, 2H), 7.88 (AA’BB’, 2H), 7.54 (d, *J* = 9 Hz, 2H), 7.38 (d, *J* = 9 Hz, 2H). ^13^C NMR (125 MHz, DMSO-*d6*) δ 164.5 (1C), 151.9 (1C), 145.7 (1C), 136.2 (1C), 132.9 (1C), 129.0 (2C), 121.9 (2C), 121.5 (2C), 120.8 (2C), 120.4 (q, *J* = 256 Hz, 1C), 120.3 (q, *J* = 257 Hz, 1C). HRMS (ESI): *m*/*z* [M + H]^+^ calculated for C_15_H_9_F_6_NO_3_: 366.0559 found: 366.0562.

*N*-Phenylbenzamide (**A4**). 1H and 13C NMR and HRMS are in agreement with previously reported data [19].

*3-[3-Chloro-5-(trifluoromethyl)phenyl]-1-[6-({[3-chloro-5-(trifluoromethyl)phenyl]carbamoyl}camino)hexyl]urea* (**D1**). ^1^H NMR (400 MHz, DMSO-*d6*) δ 8.97 (s, 2H), 7.76 (s, 2H), 7.75 (s, 2H), 7.27 (s, 2H), 6.40 (t, *J* = 5.6 Hz, 2H), 3.08 (q, *J* = 6.2 Hz, 4H), 1.44 (m, 4H), 1.29 (m, 4H). ^13^C NMR (100 MHz, DMSO-*d6*) δ 154.7 (2C), 143.0 (2C), 134.1 (2C), 130.9 (q, *J* = 32 Hz, 2C), 123.4 (q, *J* = 271 Hz, 2C), 120.3 (2C), 116.7 (2C), 112.3 (2C), 39.1 (2C), 29.6 (2C), 26.1 (2C). HRMS (ESI): *m*/*z* [M − H]^−^ calculated for C_22_H_22_Cl_2_F_6_N_4_O_2_: 557.0953 found: 557.0953.

*3-[4-(Trifluoromethyl)phenyl]-1-[6-({[4-(trifluoromethyl)phenyl]carbamoyl}amino)hexyl]urea* (**D2**). ^1^H NMR (400 MHz, DMSO-*d6*) δ 8.86 (s, 2H), 7.58 (d, *J* = 8.9 Hz, 4H), 7.54 (d, *J* = 8.9 Hz, 4H), 6.30 (t, *J* = 5.5 Hz, 2H), 3.09 (q, *J* = 6.6 Hz, 4H), 1.43 (m, 4H), 1.31 (m, 4H). ^13^C NMR (100 MHz, DMSO-*d6*) δ 154.8 (2C), 144.3 (2C), 125.9 (2C), 124.7 (q, *J* = 269 Hz, 2C), 120.8 (q, *J* = 32 Hz, 2C), 117.1 (2C), 39.0 (2C), 29.6 (2C), 26.1 (2C). HRMS (ESI): *m*/*z* [M − H]^−^ calculated for C_22_H_24_F_6_N_4_O_2_: 489.1732 found: 489.1727.

*3-[4-(Trifluoromethoxy)phenyl]-1-[6-({[4-(trifluoromethoxy)phenyl]carbamoyl}amino)hexyl]urea* (**D3**). ^1^H NMR (400 MHz, DMSO-*d6*) δ 8.60 (s, 2H), 7.47 (d, *J* = 9.2 Hz, 4H), 7.19 (d, *J* = 8.4 Hz, 4H), 6.18 (t, *J* = 5.6 Hz, 2H), 3.07 (q, *J* = 6.7 Hz, 4H), 1.43 (m, 4H), 1.30 (m, 4H). ^13^C NMR (100 MHz, DMSO-*d6*) δ 155.2 (2C), 142.0 (2C), 140.0 (2C), 121.6 (2C), 120.3 (q, *J* = 254 Hz, 2C), 118.7 (2C), 39.1 (2C), 29.7 (2C), 26.2 (2C). HRMS (ESI): *m*/*z* [M − H]^−^ calculated for C_22_H_24_F_6_N_4_O_4_: 521.1629 found: 521.1630.

*3-Phenyl-1-{6-[(phenylcarbamoyl)amino]hexyl}urea* (**D4**). 1H and 13C NMR and HRMS are in agreement with previously reported data [20].

### 2.2. Biology

#### 2.2.1. Cell Lines and Culture Conditions

Human MDA-MB-231 breast cancer cells and BEAS-2B lung epithelial cells (ATCC) were cultured in Dulbecco’s Modified Eagle Medium containing 1% (*v*/*v*) penicillin/strepotomycin (Merck, Darmstadt, Germany) and 10% (*v*/*v*) foetal bovine serum (Thermo Fisher Scientific, Waltham, MA, USA). Cells were cultured at 37 °C in a humidified atmosphere of 5% CO_2_ and harvested at 80–90% confluency using trypsin/EDTA after washing with Dulbecco’s phosphate-buffered saline (dPBS, Merck). Test compounds were administered to cells using a DMSO vehicle (final concentration 0.1% *v*/*v*) and were compared against cells treated with DMSO alone (vehicle-only control).

#### 2.2.2. MTS Cell Viability Assay

Cell viability following 72 h drug treatment was assayed by measuring the conversion of MTS (3-(4,5-dimethylthiazol-2-yl)-5-(3-carboxymethoxyphenyl)-2-(4-sulfophenyl)-2H-tetrazolium) to formazan via dehydrogenase enzymes in metabolically active cells. In a 96-well plate, cells were seeded in triplicate in complete media at a density of 3.5 × 10^3^ cells per well and incubated for 24 h. Well media were removed and replaced with complete media containing various drug concentrations and incubated for 72 h. Cells were then incubated with CellTiter MTS 96 Aqueous MTS Reagent Powder (Promega) and phenazine ethosulfate (Merck) under dark conditions for approximately 3 h. The absorbance of each well at 490 nm was measured using a Tecan Infinite M1000 Pro plate reader to evaluate cell viability (CellTiter 96 Aqueous Non-Radioactive Cell Proliferation Assay, Promega).

#### 2.2.3. JC-1 Mitochondrial Membrane Potential Assay

The ability of the test compounds to depolarise the inner mitochondrial membrane was measured using the JC-1 assay. MDA-MB-231 cells were seeded in triplicate in black wall 96-well plates (1.5 × 10^4^ cells per well) in complete media 24 h before treatment. Media were removed and cells were treated with various drug concentrations in complete media and incubated for one hour. Cells were incubated with JC-1 in media for 20 min, washed with dPBS and read using a Tecan Infinite M1000 Pro plate reader to evaluate red (535 nm) and green (595 nm) fluorescence using excitation wavelengths of 485 and 535 nm, respectively (JC-1 Mitochondrial Membrane Potential Assay Kit; Cayman Chemical).

#### 2.2.4. Seahorse XFe24 Analyser Assay

Mitochondrial function was measured by determining the oxygen consumption rate (OCR) and extracellular acidification rate (ECAR) of cells with a Seahorse XFe24 extracellular flux analyser (Seahorse Bioscience, North Billerica, MA, USA) according to the manufacturer’s protocol. MDA-MB-231 cells were seeded in Seahorse XF24 well cell culture microplates (2.0 × 10^4^ cells per well) in complete media, allowed to adhere for 3 h at room temperature, and incubated overnight. Wells were washed and media were replaced with 500 μL of XF media (1 mM pyruvate, 2 mM glutamine, and 10 mM glucose Seahorse XF DMEM, pH 7.4 with 5 mM HEPES) and placed in a non-CO_2_ incubator (37 °C, humidified) for 1 h, and then the OCR was measured utilizing an XF Cell Mito Stress Test kit (Seahorse Bioscience, MA, USA). Following sensor calibration and baseline measurements, test compounds were added and changes to the oxygen consumption rate (OCR) and extracellular acidification rate (ECAR) were monitored using a modified cycling program on Agilent Seahorse Wave Desktop software.

#### 2.2.5. Statistical Analysis

All IC_50_ concentration values are expressed as means ± SEM from three independent experiments (N = 3). Data were normalised to the DMSO vehicle control, and dose−response curves were constructed on GraphPad Prism 8 using log(inhibitor) versus response, variable slope (four parameters) nonlinear regressions. Equation: Y = Bottom + (Top − Bottom)/(1 + 100[(Log IC50-X) ∗ HillSlope)]. Absolute IC_50_ concentrations were then interpolated from these normalised curves, and the top constrained to 100%. Seahorse measurements were normalised to the OCR/ECAR of DMSO vehicle wells prior to the test compound addition.

## 3. Results and Discussion

### 3.1. Compound Library Design and Synthesis

Bisaryl ureas are comprised of a urea anion binding group covalently attached to two substituted aromatic rings. To investigate the capacity of alternative anion binding motifs to function as fatty acid-activated mitochondrial uncouplers, we designed a library of bisaryl squaramide (**S1**–**4**), amide (**A1**–**4**), and diurea (**D1**–**4**) transporters bearing various aromatic substituents (Figure 2) and compared their activities to their previously reported [11] urea counterparts, **U1**–**4**. Amides and squaramides are well-known anion binding groups that have previously been used in anion transporters [16,21,22]. Squaramides typically have stronger anion affinity than amides, as squaramides can form two convergent hydrogen bonds to host anions via their N-H groups, and have been explored using a variety of scaffolds [23]. Diureas **D1**–**4** were included as multi-armed anion transporters and can have superior anion transport to their mono-derivatives [10]. All bisaryl anion transporters were prepared as unsubstituted analogues (series **4**), and bearing lipophilic electron-withdrawing substituents, which we have previously shown to enhance fatty acid-activated proton transport and the cytotoxicity of bisaryl ureas [11]. Three different substitution patterns were included, based on electron-withdrawing strength (determined from their Hammet’s substituent constants, σ_total_) [24]. These included 3-chloro 5-trifluoromethyl- (series **1**, σ_total_ = 0.80), 4-trifluoromethyl- (series **2**, σ_total_ = 0.54) and 4-trifluoromethoxy- (series **3**, σ_total_ = 0.35) substitution.

**Scheme 1:**
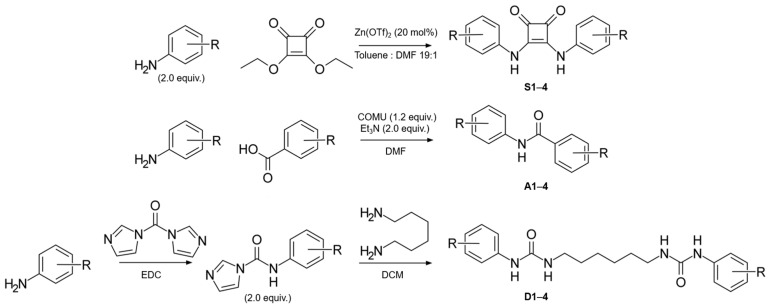
Synthesis of bisaryl squaramide (**S1**–**4**), amide (**A1**–**4**) and diurea (**D1**–**4**) anion transporters.

The synthesis of the compound library is shown in Scheme 1. Bisaryl squaramides **S1**–**4** were prepared via Lewis-acid-promoted condensation reactions of appropriately substituted anilines with squarate esters. A Zn(OTf)_2_ Lewis acid catalyst was preferred to minimise the formation of unwanted squaramide monoester and squaraine side products [25] and the squaramide products precipitated from the reaction mixture and isolated by filtration. Bisaryl amides **A1**–**4** were synthesised by COMU-based coupling reactions of the corresponding substituted anilines and benzoic acids with two equivalents of triethylamine [26]. Bisaryl diurea derivatives **D1**–**4** were synthesised by first reacting appropriately substituted anilines with *N,N*-carbonyldiimidazole (CDI) to give the corresponding *N*-carbamoylimidazoles, which were then reacted with hexamethylenediamine to give the disubstituted product. 

### 3.2. Bisaryl Anion Transporter Effects in MDA-MB-231 Cells

We first determined the ability of the bisaryl anion transporters to reduce the viability of MDA-MB-231 cells using the MTS assay (Table 1 and Figure S1). The urea-based anion transporters were the most active in the series, with **U1** and **U2** reducing MDA-MB-231 cell viability with IC_50_ concentrations below 1 μM. The corresponding squaramides **S1** and **S2** were the next most active, and reduced cell viability with IC_50_ concentrations of 4.15 ± 1.4 and 7.28 ± 0.9 μM, respectively. Within the diurea and amide series, only the di-substituted analogues **D1** and **A1** affected cell viability, although **A1** lacked sufficient activity and solubility to produce an IC_50_ concentration. Neither **D2** nor **A2** had an impact on cell viability. The MTS data also indicated that substitution with electron-withdrawing groups was required, as no unsubstituted anion transporters (series **4**) had an effect on cell viability, while those bearing the strongest electron-withdrawing substituents (series **1**) were the most active. It was also found that the MTS activity decreased as the electron-withdrawing capacity of the substituents decreased. For example, **U3** (σ_total_ = 0.35) failed to reduce cell viability to below 50% at 100 μM, while **U1** and **U2** (σ_total_ > 0.54) reduced MDA-MB-231 cell viability with IC_50_ concentrations below 1 μM. The exception to this trend was **A3**, which was the most active in the amide series, although it contains relatively weakly electron-withdrawing trifluoromethoxy substituents (series **3** substitution). It is noteworthy that **A3** did not affect mitochondrial polarisation (as reflected by the JC-1 assay), as this suggests that **A3** reduces cell viability via a different mechanism that does not involve mitochondrial targeting.

To confirm that the MTS data reflected reductions in the number of viable cells, phase contrast images of MDA-MB-231 cells treated with the active compounds **U1**, **U2**, **S1**, **S2**, **A3** and **D1** at their IC_50_ concentrations were recorded (Figure S2 in the Supplementary Material). Vehicle (DMSO) control cells and cells treated with the inactive compound **U4** were included for comparison. The images revealed that the MTS active compounds (but not inactive **U4**) reduced the number of cells and altered their morphology compared to control, which is consistent with the MTS data. We also assessed the antiproliferative activity of **U1**, **U2**, **S1**, **S2**, **A3** and **D1** against BEAS-2B lung epithelial cells in MTS assays to obtain an indication of the selectivity of these compounds towards cancer cells. As shown in Figure S3 of the Supplementary Material, **U1**, **U2** and **S1** preferentially reduced MDA-MB-231 cell viability, while **A3** had no effect on BEAS-2B cells. In contrast, diurea **D1** and squaramide **S2** had similar effects on the viability of both cell lines.

We next assessed the capacity of the analogues to transport protons across the MIM and depolarise mitochondria in MDA-MB-231 cells using JC-1 assays. JC-1 is a cationic, fluorescent dye that partitions between the mitochondrial matrix and the cytosol according to the Δ_ΨM_. In polarised mitochondria with high Δ_ΨM_, JC-1 forms aggregates in the matrix that fluoresce red. When mitochondrial depolarisation occurs, JC-1 is released into the cytosol and disaggregates into monomers that fluoresce green. Thus, the JC-1 red/green fluorescence ratio reflects the proton gradient across the MIM. JC-1 IC_50_ concentrations were determined for the bisaryl analogues from dose–response curves (Figure S4), which were defined as the concentration required to shift the red/green fluorescence ratio by 50% of the control (Table 1). Cells were treated for one hour to discriminate between direct mitochondrial uncoupling activity and the mitochondrial depolarisation that occurs during apoptosis.

Similar to the trends observed from the MTS data, the urea-based **U1** was the most active (IC_50_ = 0.26 ± 0.1) anion transporter with series **1** substitution, followed by squaramide **S1** (IC_50_ = 1.73 ± 0.1), and then the amide and diurea analogues **A1** and **D1**. The dependence on electron-withdrawing substituents for activity was also observed, as JC-1 IC_50_ concentrations tended to increase moving from series **1** to series **2** to series **3** substitution patterns, with all unsubstituted analogues (series **4**) lacking sufficient JC-1 activity to determine IC_50_ concentrations. The broad similarities between the observed MTS and JC-1 data indicate that the active anion transporters depolarise mitochondria, which leads to a loss of cell viability. This finding is consistent with previous studies, which have shown that the collapse of the proton gradient across the MIM by mitochondrial uncouplers leads to cancer cell death [9,11,23].

**Figure 3:**
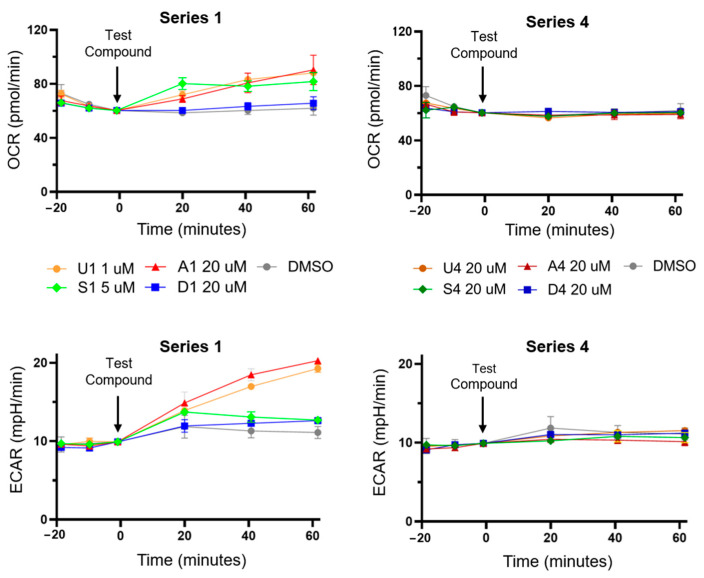
OCR and ECAR in MDA-MB-231 cells treated with series **1** and **4** test compounds as measured using a Seahorse XFe24 flux analyser. Measurements normalised to the OCR and ECAR of DMSO vehicle wells prior to test compound addition.

To further investigate the mitochondrial actions of the anion transporters, we measured the capacity of series **1** (3-Cl 5-CF_3_) and **4** (unsubstituted) analogues to increase the oxygen consumption rate (OCR) and extracellular acidification rate (ECAR) of MDA-MB-231 cells using a seahorse analyser (see Figure 3). Proton transport across the MIM by mitochondrial uncouplers reduces OXPHOS efficiency, leading to an increase in oxygen consumption and a shift to glycolysis (proportional to ECAR) to meet energy demands [6]. Anion transporters that were JC-1 active were anticipated to produce the same metabolic shifts, and were tested at concentrations two-fold higher than their JC-1 IC_50_ concentrations.

As anticipated, the JC-1 active anion transporters **U1**, **S1** and **A1** increased OCR and ECAR in MDA-MD-231 cells, which is consistent with their activity as mitochondrial uncouplers, and JC-1 inactive anion transporters **U4**, **S4**, **A4** and **D4** failed to impact OCR and ECAR (Figure 3). Diurea **D1** had the smallest impact on OCR and ECAR, which may reflect its lack of potency in JC-1 assays.

### 3.3. HPTS Proton Transport Assay

The cellular data suggest that bisaryl anion transporters substituted with electron withdrawing substituents (series **1**) can transport protons across the MIM in MDA-MB-231 cells, leading to mitochondrial uncoupling and a loss of cell viability, while unsubstituted (series **4**) transporters lack activity. We therefore studied the proton transport mechanisms of the series **1** and **4** anion transporters using the cell-free 8-hydroxy-1,3,6-pyrene trisulfonic acid (HPTS) proton transport assay to identify the underlying proton transport mechanisms [9]. Our previous work determined that bisaryl urea **U1** operated predominantly via the fatty acid-facilitated proton transport mechanism rather than directly cycling protons in a unimolecular process. The strong binding affinity of the urea group towards the carboxylate head groups, and the efficient shuttling across the membrane due to the presence of multiple lipophilic groups, were noted as contributing factors to its high proton transport activity [11].

Large unilamellar 1-palmitoyl-2-oleoyl-*sn*-glycero-3-phosphocholine vesicles (POPC, 200 nm) were prepared in a solution of potassium gluconate (100 mM) and HEPES buffer (10 mM). HPTS, a pH-sensitive fluorescent probe, was encapsulated inside the vesicles and used to measure the intravesicular pH (see Supplementary Material for full details) [27]. Once a pH gradient had been established by the addition of a base pulse to the extravesicular medium, dissipation across the membrane occurred via proton transport facilitated by the target compound.

**Figure 4:**
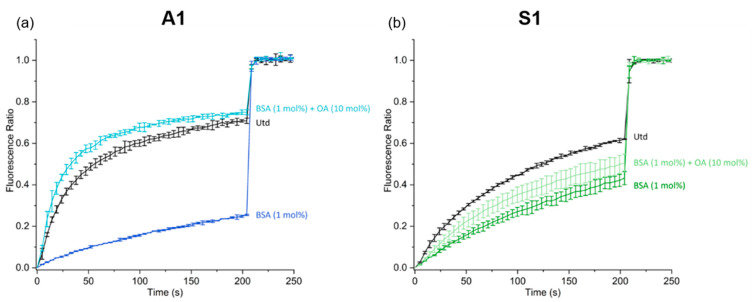
(**a**) The proton transport induced in the HPTS assay by **A1** at a molar-to-lipid concentration of 0.1 mol%, under various experimental conditions. Black represents untreated vesicles, dark blue represents BSA-treated vesicles, and light blue represents OA-treated vesicles. (**b**) The proton transport induced in the HPTS assay by **S1** at a molar-to-lipid concentration of 0.02 mol%, under various experimental conditions. Black represents untreated vesicles, dark green represents BSA-treated vesicles, and light green represents OA-treated vesicles.

Initially, dose–response studies were conducted for each compound in untreated POPC vesicles to establish their proton transport activity. Synthetic POPC contains trace amounts of fatty acid impurities (palmitic and oleic acid), which means that transport under these conditions may be partially facilitated by the fatty acid flip-flop mechanism [28,29]. Therefore, the studies were then repeated with vesicles treated with bovine serum albumin (BSA, 1 mol%). This protein binds to free fatty acids present in the membrane and removes them, meaning that proton transport under these conditions is a consequence of direct proton cycling, akin to classical protonophores such as CCCP [5]. The fatty acid impurities present in synthetic POPC are found at a significantly lower concentration than the quantity of BSA added to the vesicles, meaning we can safely assume that all fatty acid impurities are removed upon treatment with BSA. A final set of studies involved the addition of oleic acid (OA, 10 mol%) to the BSA-treated vesicles, which saturated the remaining BSA binding sites and resulted in a 4 mol% concentration of fatty acid present in the membrane (see Figure 4 for representative plots). An enhancement in transport activity under these conditions, compared to the results of the BSA-treated vesicles, can be attributed to proton transport facilitated by a fatty acid-activated mechanism. Transport activity was quantified by fitting the dose–response curve to an adapted Hill equation and calculating an EC_50_ value (the transport concentration required to facilitate 50% proton efflux after 200 s) under each of the experimental conditions. The Hill coefficient, *n*, provides an indication of the transporter/anion stoichiometry of the transmembrane transport event. These values, under each of the experimental conditions, are presented in Table 2**.**

In general, the results of the studies on untreated POPC vesicles correlate well with the in vitro findings. **U1** was the most active protonophore in the series (EC_50_ = 0.0046 ± 4 × 10^−4^), followed by squaramide **S1**, amide **A1** and then diurea **D1**. This same ordering of activities was observed in MTS and JC-1 assays. Unsubstituted anion transporters **U4**, **A4** and **D4** were inactive in the HPTS assay, and also lacked MTS and JC-1 activity. Squaramide **S4** was the only unsubstituted derivative to display transport activity; however, it was approximately 21 times less active than substituted squaramide **S1**, reflecting the poor electron-withdrawing ability of this substituent. Compounds bearing the series **4** substitution were less likely to bind to carboxylate groups and facilitate fatty acid-facilitated transport, or acidify the hydrogen donor effectively enough to enable direct proton cycling. The low proton transport capacity of **S4** likely accounts for its lack of significant activity in cell-based assays.

An activation factor was calculated for each transport by dividing the EC_50_ value of the BSA-treated vesicles by the EC_50_ value of the OA-treated vesicles. A value greater than 1.0 reflects an enhancement in transport activity in the presence of free fatty acids, and that a fatty acid-facilitated mechanism is the predominant mode of proton transport. **A1** and **D1** returned activation factors of 6.00 and 3.75, respectively. These were lower than the activation factor of **U1**, but still suggest that binding to carboxylate head groups is a key mechanism of proton transport. Interestingly, the Hill coefficients for these compounds suggest a transport stoichiometry which matches a fatty acid-facilitated process. The *n* values for **A1** were all greater than 1.0, and a value of exactly *n* = 2.00 was reported in the studies using untreated vesicles. This suggests that amide donor motifs from two independent molecules were involved in binding to a single carboxylate group to facilitate its movement across the membrane. The *n* values for **D1** were less than 1.0, and *n* was close to 0.50 in the case of the OA-treated vesicles. This stoichiometry reflects a potential transport pathway where the two urea groups of a single unit of **D1** coordinate to two separate carboxylate head groups and facilitate their transport simultaneously. Proton transport purely via the classical proton cycling pathway would return a Hill coefficient closer to 1.0.

The only unsubstituted anion transporter with HPTS activity was **S4**, and the activation factor suggested that a fatty acid-facilitated mechanism was the active pathway. This may be due to the enhanced hydrogen bond donor capacity of squaramide protons, the shape-match of this motif with Y-shaped anions like carboxylates, and the smaller bite-angle of this donor leading to greater contributions to binding by the *ortho*-CH protons [30]. With these properties under consideration, we would therefore expect to see enhanced fatty acid-facilitated transport for **S1** compared to **U1**. However, **S1** in untreated POPC vesicles is 1.5 times less active than its urea analogue. Moreover, **S1** returned the lowest activation factor of the series, and instead exhibited the greatest potency in BSA-treated vesicles (Figure 4), indicating that a fatty acid-facilitated mechanism is not the preferred mechanism of proton transport for this squaramide. It is likely that the coordination of this molecule to carboxylate head groups is very strong, resulting in a reduced tendency to decomplex after the translocation of the carboxylate through the membrane—a vital step in the fatty acid “flip-flop” cycle depicted in Figure 1. Furthermore, the enhanced acidity of squaramides compared to urea groups and the delocalisation of the charge around the squarate system, coupled with the increase in lipophilicity provided by the bis-trifluoromethyl groups, means that compound **S1** can most likely facilitate proton transport by a classical proton cycling mechanism. This would account for the low activation factor calculated for compound **S1**.

The results of these experiments verify that a number of hydrogen bond donor motifs can facilitate proton transport via a fatty acid-activated mechanism. The anion transporters with electron-withdrawing substituents (series **1**) all demonstrated enhanced activity over their unsubstituted analogues; however, the results for compound **S1** also highlighted that binding to head groups can sometimes be too strong to effectively initiate the “flip-flop” cycle. This means that a combination of hydrogen bond donor species and aryl substituents must be considered to maximise proton transport via this pathway.

## 4. Conclusions

In this paper, we replaced the anion binding motif of bisaryl urea mitochondrial uncouplers with squaramide, amide and diurea groups, and investigated the capacity of these compounds to uncouple mitochondria via fatty acid-activated proton transport. Bisaryl squaramide-based anion transporters were found to uncouple mitochondria and reduce MDA-MB-231 cell viability with similar potency to their urea counterparts, while amide and diurea scaffolds were less active. LUV studies found that lipophilic electron-withdrawing groups on the amide and diurea scaffolds were essential for fatty acid-activated proton transport. However, the same substituents reduced fatty acid dependency in bisaryl squaramides, instead favouring proton transport as a classical protonophore via direct deprotonation.

## Figures and Tables

**Table 1:** Chemical structures, MTS (72 h) and JC-1 (1 h) IC_50_ concentrations measured in MDA-MB-231 breast cancer cells.

		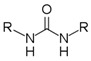 U1–4		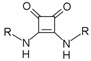 S1–4		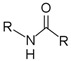 A1–4		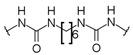 D1–4	
Substitution Pattern	R	IC_50_ (μM)		IC_50_ (μM)		IC_50_ (μM)		IC_50_ (μM)	
		MTS	JC-1	MTS	JC-1	MTS	JC-1	MTS	JC-1
Series 1	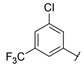	0.37 ± 0.1	0.26 ± 0.1	4.15 ± 1.4	1.73 ± 0.1	>20 ^a^	7.30 ± 1.0	10.5 ± 1.1	8.1 ± 1.2
Series 2	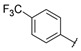	0.84 ± 0.1	2.26 ± 0.4	7.28 ± 0.9	3.34 ± 0.9	- ^b^	19.7 ± 5.9	- ^b^	>50 ^a^
Series 3	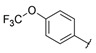	>100 ^a^	>100 ^a^	- ^b^	- ^b^	5.64 ± 1.1	- ^b^	- ^b^	>50 ^a^
Series 4	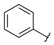	- ^b^	>100 ^a^	- ^b^	- ^b^	- ^b^	- ^b^	- ^b^	>100 ^a^

^a^ Produced moderate activity at maximum test concentration but insufficient for absolute IC_50_ determination. ^b^ No significant activity observed at the maximum test concentration.

**Table 2:** HPTS (200 s) EC_50_ concentrations and hill coefficients in untreated (Utd) POPC vesicles, BSA-treated POPC vesicles and OA-treated vesicles.

Compound ^a^	EC_50_ (Utd, mol%)	n (Utd)	EC_50_ (BSA, mol%)	n (BSA)	EC_50_ (OA, mol%)	n (OA)	Activation Factor
**U1**	0.0046 ± 4 × 10^−4^	0.82 ± 0.07	0.06 ± 0.004	1.24 ± 0.09	0.003 ± 1 × 10^−4^	0.8 ± 0.04	20
**A1**	0.02 ± 2 × 10^−4^	2.00 ± 0.07	0.12 ± 0.005	1.74 ± 0.08	0.02 ± 0.001	1.44 ± 0.2	6
**D1**	0.04 ± 0.003	0.91 ± 0.08	0.15 ± 0.02	0.58 ± 0.05	0.04 ± 0.001	0.66 ± 0.05	3.75
**S1**	0.007 ± 6 × 10^−5^	1.63 ± 0.02	0.02 ± 0.001	1.38 ± 0.11	0.01 ± 0.001	1.24 ± 0.01	2
**S4**	0.15 ± 0.006	1.09 ± 0.09	0.89 ± 0.009	1.67 ± 0.05	0.13 ± 0.008	0.74 ± 0.04	6.85

^a^ Anion transporters **U4**, **A4** and **D4** lacked activity in HPTS assays and are not shown.

## Data Availability

All data are available in the main text or the Supplementary Materials.

## Supplementary Materials

The following supporting information can be downloaded at: https://www.mdpi.com/article/10.3390/biom13081202/s1. Table S1. Library absolute quantitative ^1^H NMR purity determination; Figure S1. Representative MTS dose–response curves; Figure S2. MDA-MB-231 cell images; Figure S3. MDA-MB-231 and BEAS-2B MTS data; Figure S4. Representative JC-1 dose–response curves; Figures S5–S28. Library ^1^H and ^13^C NMR spectra; Figures S29–S41: HPTS dose–response and Hill plot analyses.
