# Supplementary material for: Fatty Acid-Activated Proton Transport by Bisaryl Anion Transporters Depolarises Mitochondria and Reduces the Viability of MDA-MB-231 Breast Cancer Cells
Source: Biomolecules. 2023 Jul 31;13(8):1202. doi: 10.3390/biom13081202 (PMC10452527; doi:10.3390/biom13081202)
Supplement: Supplementary file 1 [file biomolecules-13-01202-s001.zip › biomolecules-2468051-supplementary.pdf]

biomolecules

MDPI

Supplementary Information

Fatty Acid-Activated Proton Transport by Bisaryl Anion Transporters Depolarises Mitochondria and Reduces the Viability of MDA-MB-231 Breast Cancer Cells

Edward York [1], Daniel A. McNaughton [1,2], Meryem-Nur Duman [1], Philip A. Gale [1,2,3] and Tristan Rawling [1,*]

[1] School of Mathematical and Physical Sciences, Faculty of Science, University of Technology Sydney, Sydney, NSW 2007, Australia
[2] School of Chemistry, The University of Sydney, Sydney, NSW 2006, Australia
[3] The University of Sydney Nano Institute (SydneyNano), The University of Sydney, Sydney, NSW 2006, Australia
* Correspondence: Tristan.Rawling@uts.edu.au

Table of Contents

Table of Contents 1

1.1 H+/OH⁻ Transport Assay in Vesicles 2

1.2 Absolute Quantitative ^{1}H NMR Purity Determination of S1-4, A1-4 and D1-4 4

1.3 Representative MTS Dose–Response Curves 5

1.4 Images of MDA-MB-231 Cells Treated with U1, U2, U4, S1, S2, A3 and D1 6

1.5 Viability of MDA-MB-231 and BEAS-2B Cells Treated with U1, U2, S1, S2, A3 and D1 7

1.6 Representative JC-1 Dose–Response Curves 8

1.7 ^{1}H NMR and ^{13}C NMR Spectra 9

1.8 S1/S4, A1/A4, D1/D4 HPTS Dose–Response Hill Analyses 21

References 27

1.1. H^+/OH^- Transport Assay in Vesicles

Vesicle Preparation

HPTS assays were conducted using POPC LUVs (200 nm diameter) vesicles loaded with an internal solution containing pH-sensitive fluorescent dye HPTS (1 mM), HEPES buffer (10 mM) and potassium gluconate (100 mM). An external solution of HEPES buffer (10 mM) and potassium gluconate (100 mM) was also prepared, and both solutions were buffered to pH 7.

Unilamellar vesicles were prepared following a procedure outlined previously by the Gale group [27]. A chloroform solution of POPC (37.5 mM, 4 mL) was transferred to a preweighed round-bottomed flask, and the solvent was removed using a rotary evaporator. The pressure was lowered slowly to ensure the formation of a smooth lipid film. Subsequently, the film was dried in vacuo for 4–24 h, and the mass of lipid was recorded. The lipids were rehydrated with 4 mL of internal solution (this number should correspond to the volume of POPC solution used initially) and vortexed until all lipids were removed from the sides of the flask and were suspended in solution. The lipids were subjected to nine cycles of freeze–thaw by freezing using a dry ice/acetone bath and thawing in lukewarm water. Following this, the vesicles were left to rest at room temperature for 30 min. The lipids were extruded through a 200 nm polycarbonate membrane 25 times to form monodisperse vesicles. Only 1 mL of solution was extruded at a time before being collected. Finally, any residual unencapsulated salt from the internal solution was removed using a B19 column packed with hydrated G-25 Sephadex®, which had been pre-saturated with the respective external solution. The lipid suspensions were diluted with the external solution to afford a stock solution (10 mL) of a known concentration.

When fatty acid removal by BSA was required, fatty-acid-free BSA was dissolved in the vesicle stock suspension to a final BSA concentration of 1 mol% (with respect to lipids).

The BSA-containing vesicle stock suspension was stirred for 20 min before being used for membrane transport studies.

HPTS Assay

For a given experiment, the prepared vesicles were diluted to a concentration of 0.1 mM in a 4.5 mL plastic cuvette. A pH gradient was required to drive transport through the vesicle membrane in these experiments before the transporter was added. An aliquot of aqueous KOH solution (25 μL, 0.5 M) was added to increase the pH of the external solution by approx. one pH unit, to pH 8.0. For experiments requiring the addition of Oleic Acid (5 μL of 5 mM DMSO solution, 10 mol%, corresponding to ~4 mol% free concentration after BSA binding), it was added before KOH. Following this, valinomycin (5 μL of 25 μM DMSO solution, 0.05 mol%) was added to each cuvette. Transport was initiated with the addition of the transporter as a DMSO solution (5 μL) and ended with the addition of detergent (Triton X-100 (10% v/v in water), 25 μL) which was added at t = 205 s to lyse the vesicles, and a final fluorescence intensity reading was recorded at t = 300 s to signify 100% proton efflux.

Experiments were conducted under three conditions: i) untreated vesicles (KOH and valinomycin addition); ii) BSA-treated vesicles (KOH and valinomycin addition); and iii) BSAtreated vesicles with OA addition (OA, KOH and valinomycin addition).

Dose–Response Hill Analysis

The changes in the fluorescent activity of intravesicular HPTS were used to detect pH changes during the experiments, and hence represent the proton efflux. The acidic and basic forms of the HPTS probe were excited at λex = 403 nm and λex = 460 nm, respectively, and the fluorescence emission of both forms was recorded at λem = 510 nm. The intensity ratio of the basic form to acidic form was determined, and the fractional fluorescence intensity (IF) was calculated using the equation:

$$I_F = \frac{R_t - R_0}{R_d - R_0}$$

where R_t is the ratiometric fluorescence value at a given time (t), R_0 is the ratiometric fluorescence value at t = 0 s and R_d is the fluorescence ratiometric value recorded at t = 280 s following the addition of detergent.

Dose–response experiments were performed at a minimum of five transporter concentrations plus a blank DMSO control run. The fractional fluorescence intensity (I_F) was plotted as a function of transporter concentration (mol%, with respect to lipid concentration). The I_F value at t = 200 s for each tested transporter concentration was fitted to an adapted Hill Equation using Origin 2021b (Academic), given as:

$$y = y_0 + (y_{max} - y_0)\frac{x^n}{k^n + x^n}$$

where y_0 is the I_F value at t = 200 s for the DMSO blank run, y_{max} is the maximum I_F value, n is the Hill coefficient and k is a derived parameter.

A derived equation was used to calculate the EC_{50} value, the transporter concentration required to facilitate 50% proton efflux, given as:

$$EC_{50} = k\left(\frac{0.5}{y_1 - y_0}\right)^{1/n}$$

where k and n are the derived parameters from the Hill equation, y_0 is the percentage proton efflux at t = 0 s and y_1 is the percentage proton efflux at t = 280 s.

1.2. Absolute quantitative ^{1}H NMR purity determination of S1-4, A1-4 and D1-4

The purity of all test compounds was confirmed to be >95% using absolute quantitative NMR spectroscopy according to the protocol outlined by the Journal of Medicinal Chemistry [17]. This technique determines purity by comparing the NMR signals of a given analyte against an internal calibrant (IC) of known purity. DMSO-d6 was spiked with a known mass of the internal calibrant, 1,3,5-trioxane (99.5% purity), to give a final IC concentration of approximately 1.5 mg/ml. Approximately 5 mg of each test compound was accurately weighed on a five-decimal place analytical balance (0.01 mg accuracy). The sample was then dissolved in 600 μL of IC-spiked DMSO-d6 administered via mechanical pipette, then transferred to 5 mm NMR tubes. The collection and processing of NMR spectra, and the calculation of purity, were performed according to the procedure described by the Journal of Medicinal Chemistry. The final calculated purities are shown in Table S1.

Table S1. Bisaryl anion transporter qNMR table.

Copyright: © 2023 by the authors. Licensee MDPI, Basel, Switzerland. This article is an open access article distributed under the terms and conditions of the Creative Commons Attribution (CC BY) license (https://creativecommons.org/licenses/by/4.0/).

**Compound**	**Purity (%)**
S1	98.0
S2	100.5
S3	99
S4	96.5
A1	99.4
A2	100.6
A3	100.6
A4	100.6
D1	97.1
D2	95.3
D3	99.7
D4	96.8

1.3. Representative MTS Dose–Response Curves

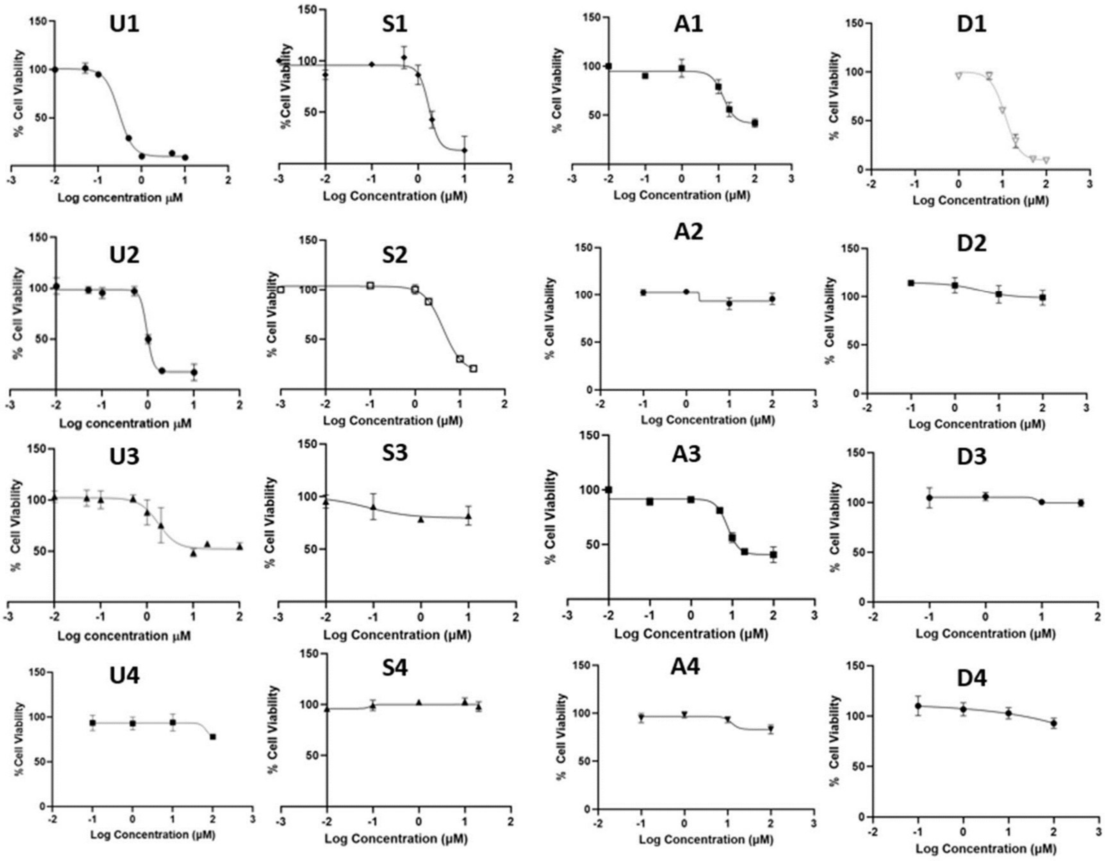

Figure S1. Representative dose–response curves showing the antiproliferative effects of U14, S1-4, A1-4 and D1-4 in MDA-MB-231 cells in a 72 h MTS cell viability assay. Dose–response curves were constructed using log(inhibitor) vs. response, variable slope (4 parameters) nonlinear regressions on GraphPad Prism 8. Absolute IC_{50} concentrations were interpolated from these normalised curves (data normalised to DMSO vehicle control) with the top constrained to 100%. Equation: Y=Bottom + (Top-Bottom)/(1+10^((LogIC50X)*HillSlope)).

1.4. Images of MDA-MB-231 Cells Treated with U1, U2, U4, S1, S2, A3 and D1

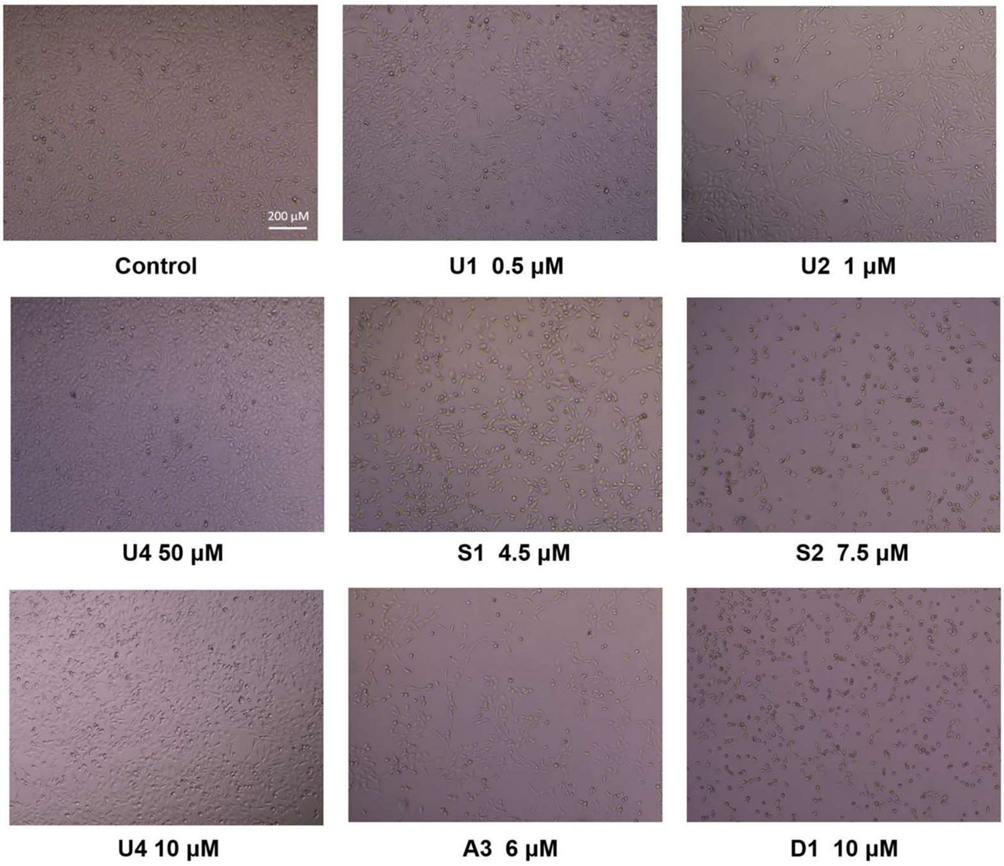

Figure S2. Images of MDA-MB-231 cells treated with U1, U2, S1, S2, A3 and D1 for 72 hours at the concentrations indicated. Cells treated with the inactive compound U4 (10 µM, 50 µM) and vehicle (DMSO) only are shown for comparison. Cells were seeded in 12-well plates (2.5 x 10^4 cells/well) and imaged following treatment at 40X magnification using a Lumenera Infinity 1-2CB microscope camera.

1.5. Viability of MDA-MB-231 and BEAS-2B Cells Treated with U1, U2, S1, S2, A3 and D1

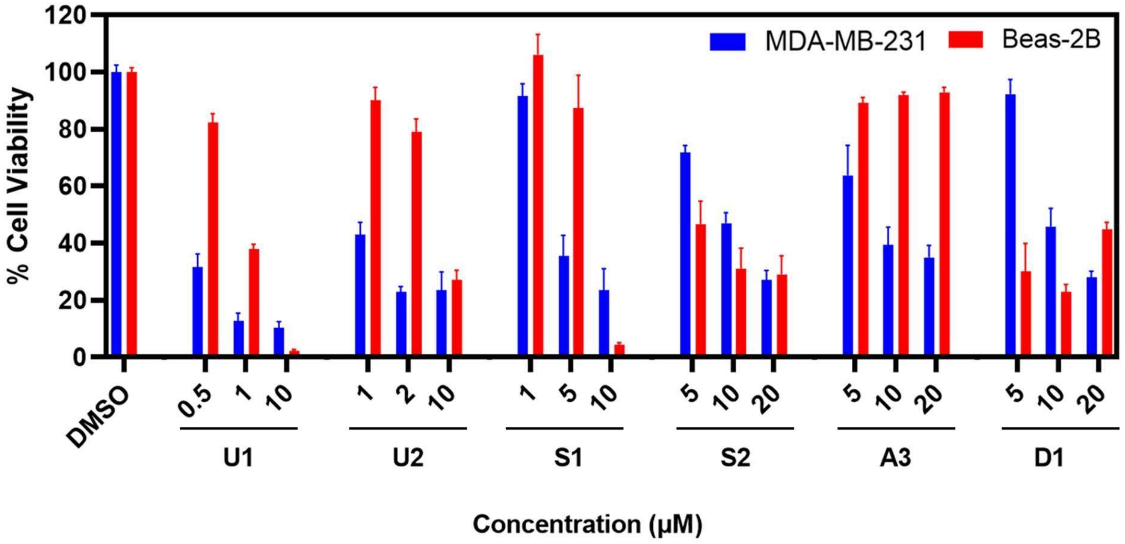

Figure S3. Effects of U1, U2, S1, S2, A3 and D1 on MDA-MB-231 and BEAS-2B cell viability measured by 72 h MTS assays. Compounds with MTS activity in MDA-MB-231 cells were tested in BEAS-2B cells at three common concentrations near and above their corresponding IC_{50} concentrations to indicate selectivity between cell lines. Beas-2B MTS assays were performed as described in the main text.

1.6. Representative JC-1 Dose–Response Curves

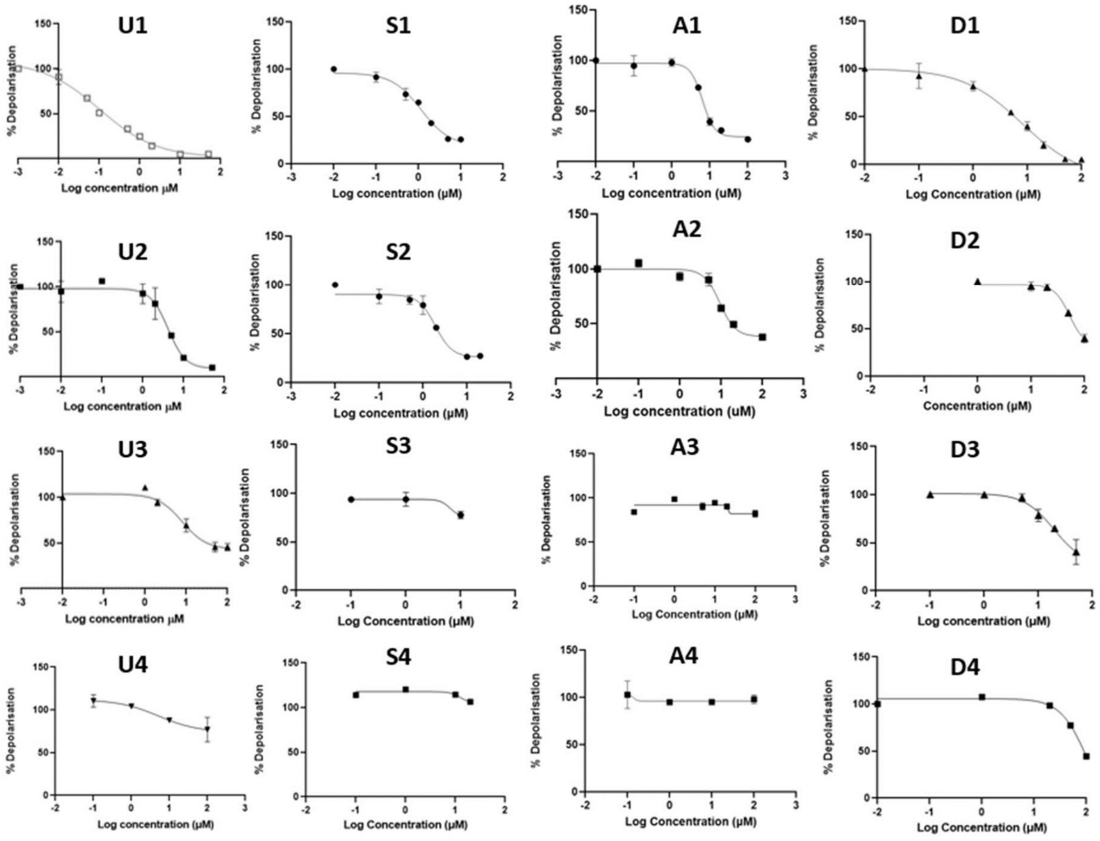

Figure S4. Representative dose–response curves showing the effects of U1-4, S1-4, A1-4 and D1-4 on the JC-1 red/green fluorescence ratio in MDA-MB-231 breast cancer cells after 1 hour of treatment with the test compound. Dose–response curves were constructed using log(inhibitor) vs. response, variable slope (4 parameters) nonlinear regressions on GraphPad Prism 8. Absolute IC50 concentrations were interpolated from these normalised curves (data normalised to DMSO vehicle control) with the top constrained to 100%. Equation: Y=Bottom + (Top-Bottom)/(1+10^((LogIC50-X)*HillSlope)).

1.7. H NMR and ^{13}C NMR Spectra

Bis({[3-chloro-5-(trifluoromethyl)phenyl]amino})cyclobut-3-ene-1,2-dione (S1).

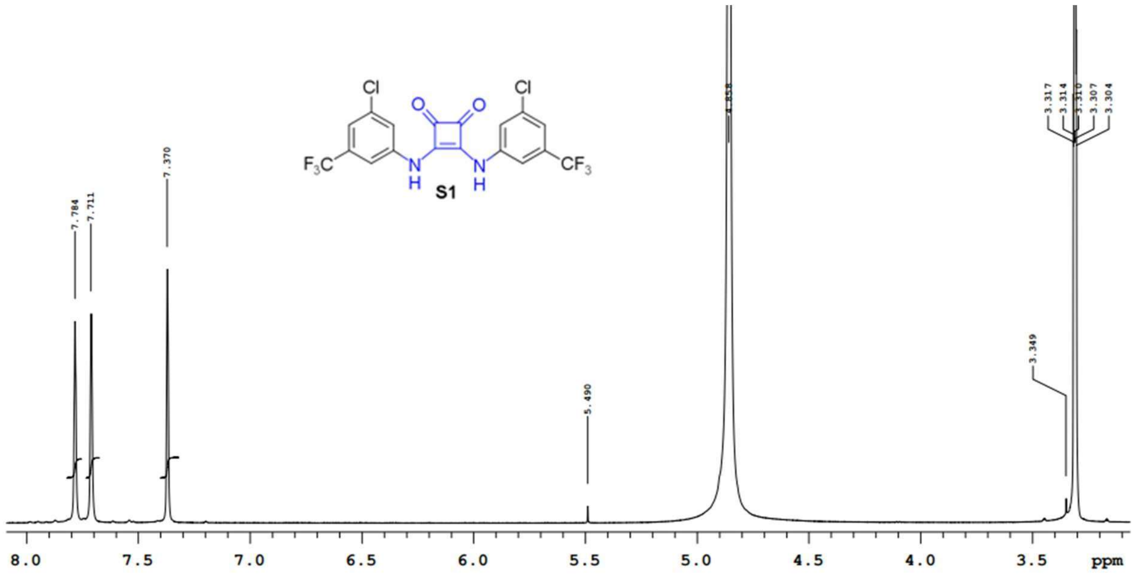

Figure S5. The 500 MHz ^{1}H NMR spectrum of S1.

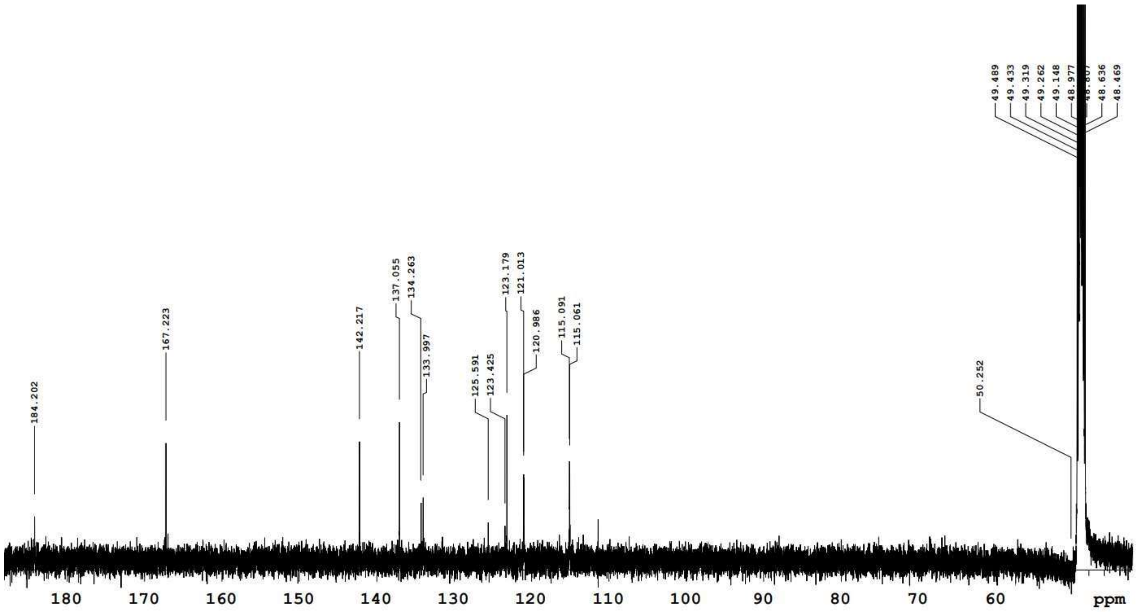

Figure S6. The 125 MHz ^{13}C NMR spectrum of S1.

Bis({[4-(trifluoromethyl)phenyl]amino})cyclobut-3-ene-1,2-dione (S2).

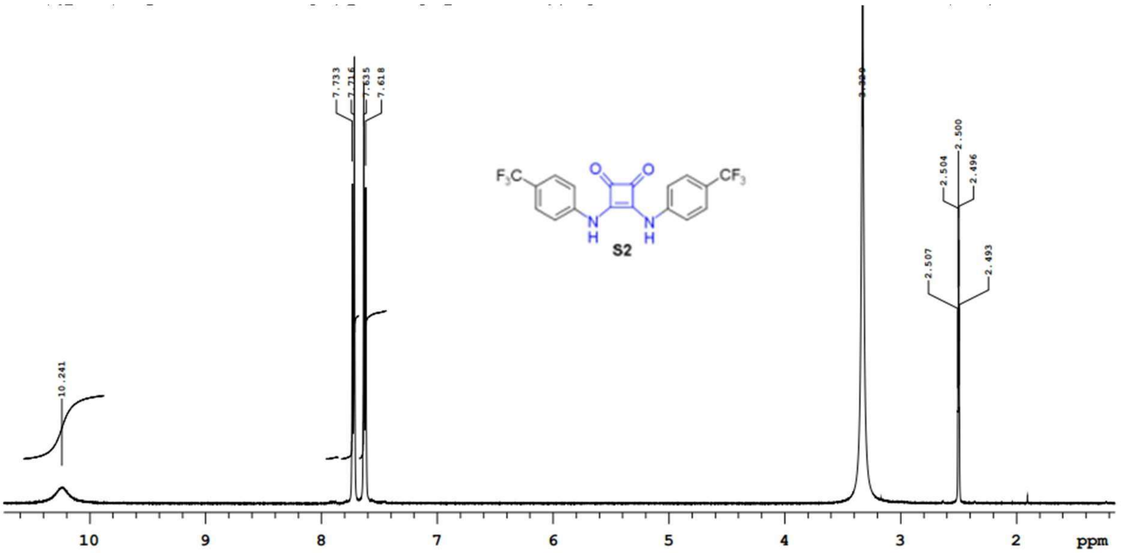

Figure S7. The 500 MHz ^{1}H NMR spectrum of S2.

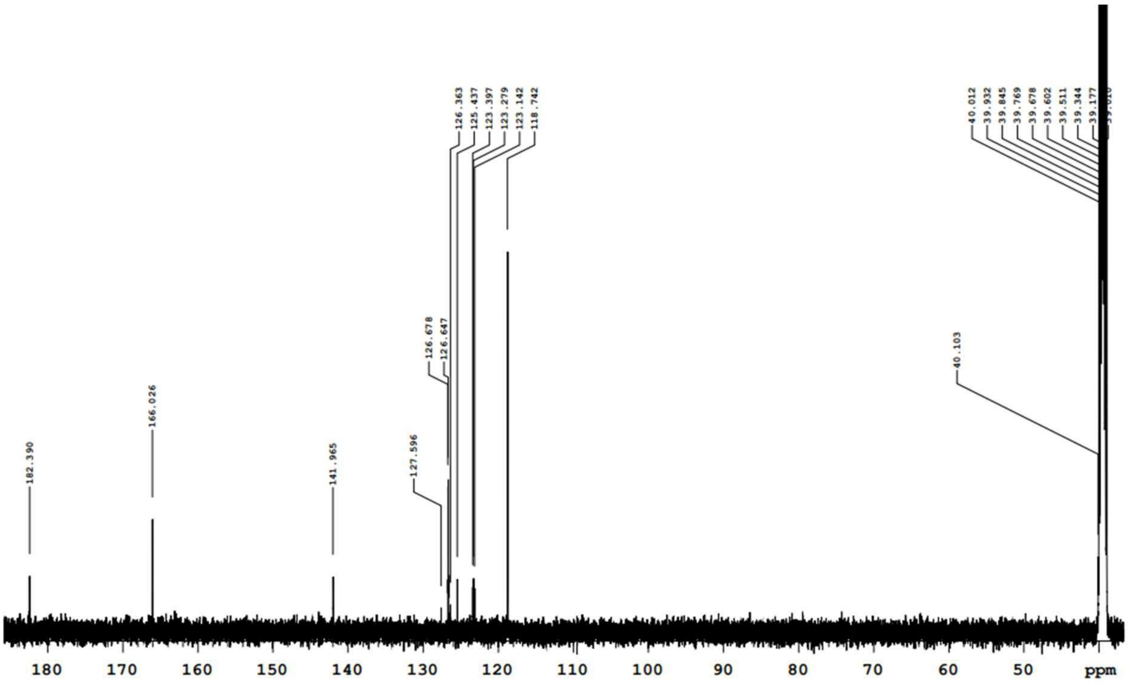

Figure S8. The 125 MHz ^{13}C NMR spectrum of S2.

Bis({[4-(trifluoromethoxy)phenyl]amino})cyclobut-3-ene-1,2-dione (S3).

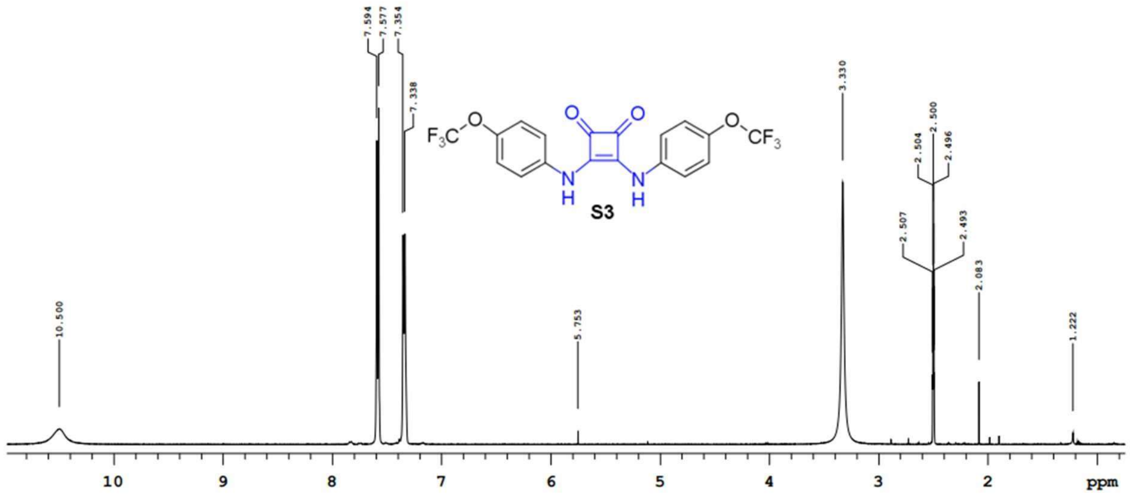

Figure S9. The 500 MHz ^{1}H NMR spectrum of S3.

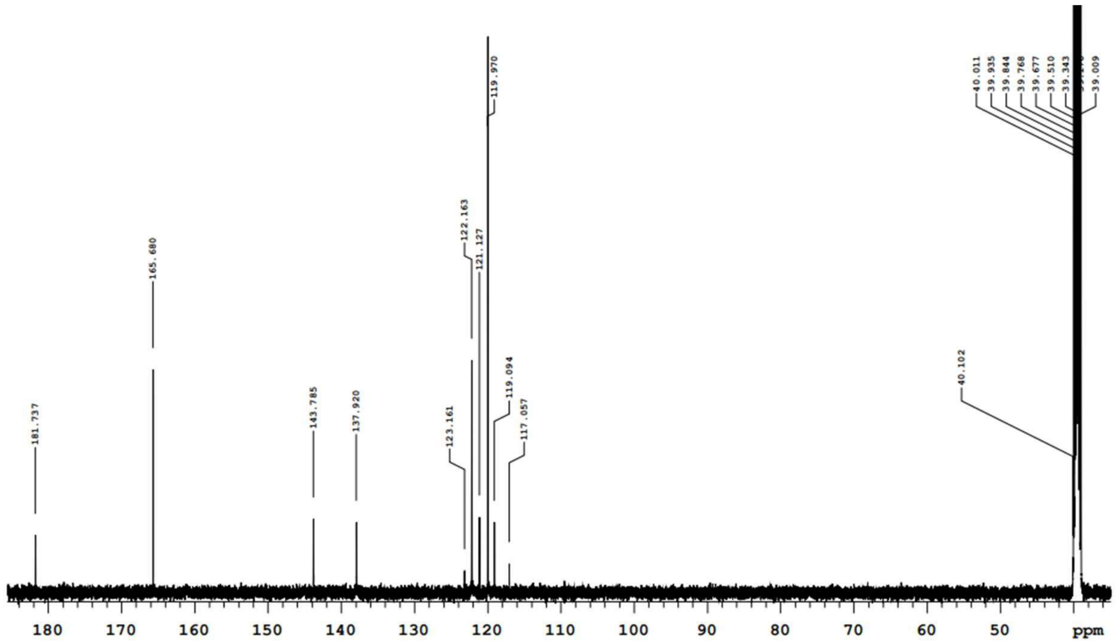

Figure S10. The 125 MHz ^{13}C NMR spectrum of S3.

Bis(phenylamino)cyclobut-3-ene-1,2-dione (S4).

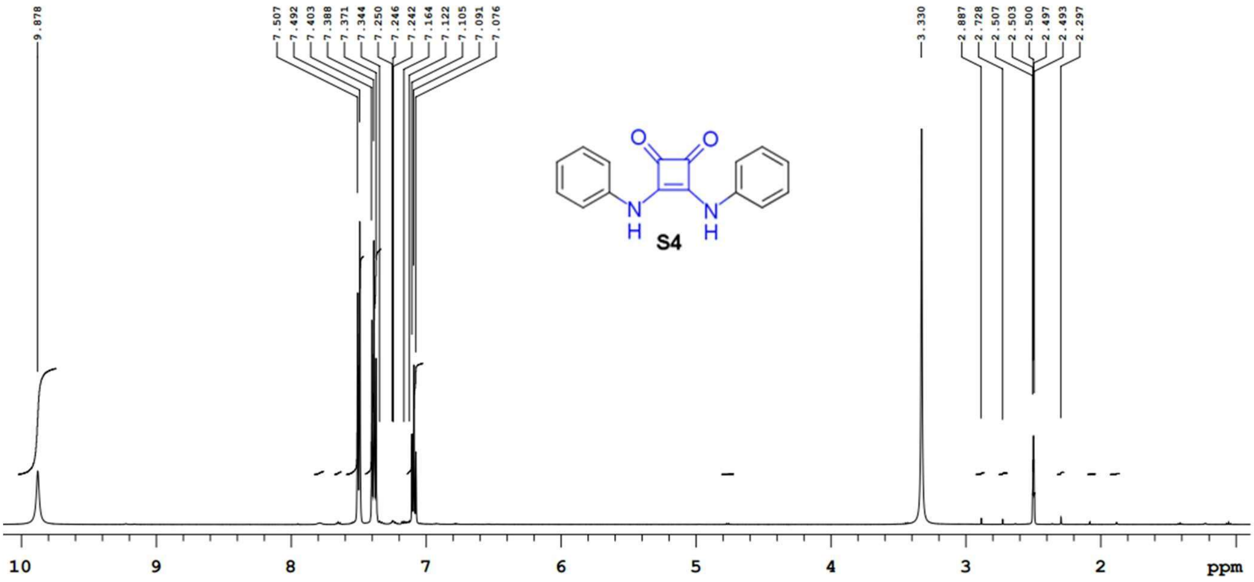

Figure S11. The 500 MHz ^{1}H NMR spectrum of S4.

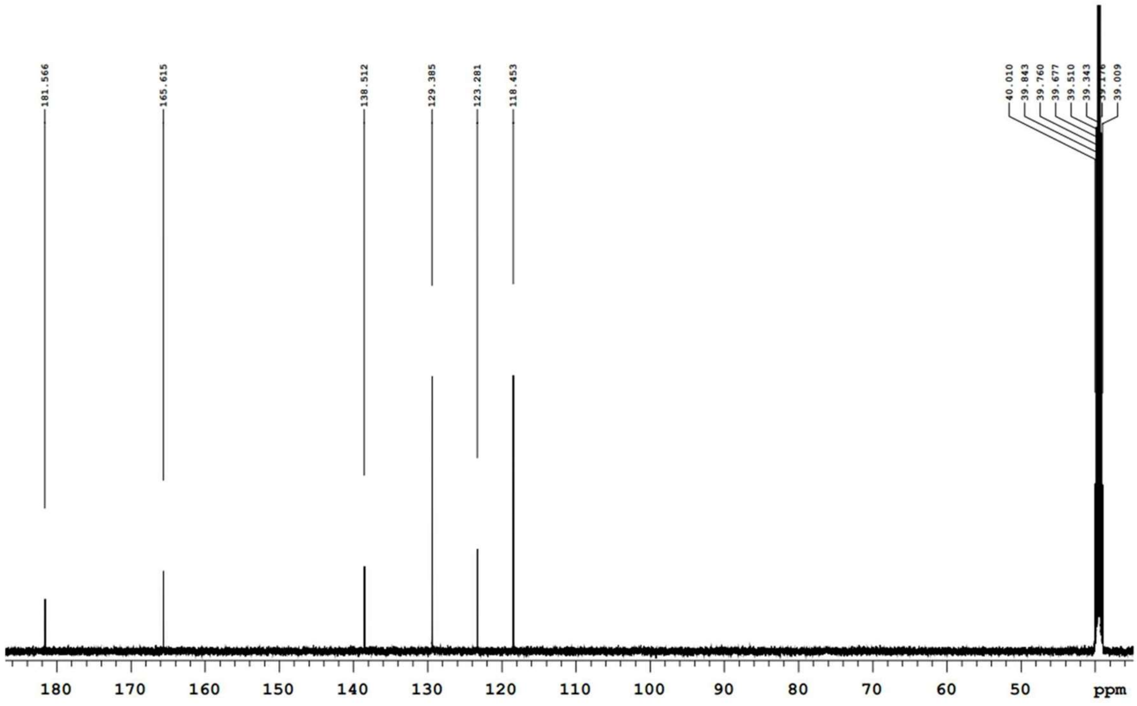

Figure S12. The 125 MHz ^{13}C NMR spectrum of S4.

3-chloro-N-[3-chloro-5-(trifluoromethyl)phenyl]-5(trifluoromethyl)benzamide (A1).

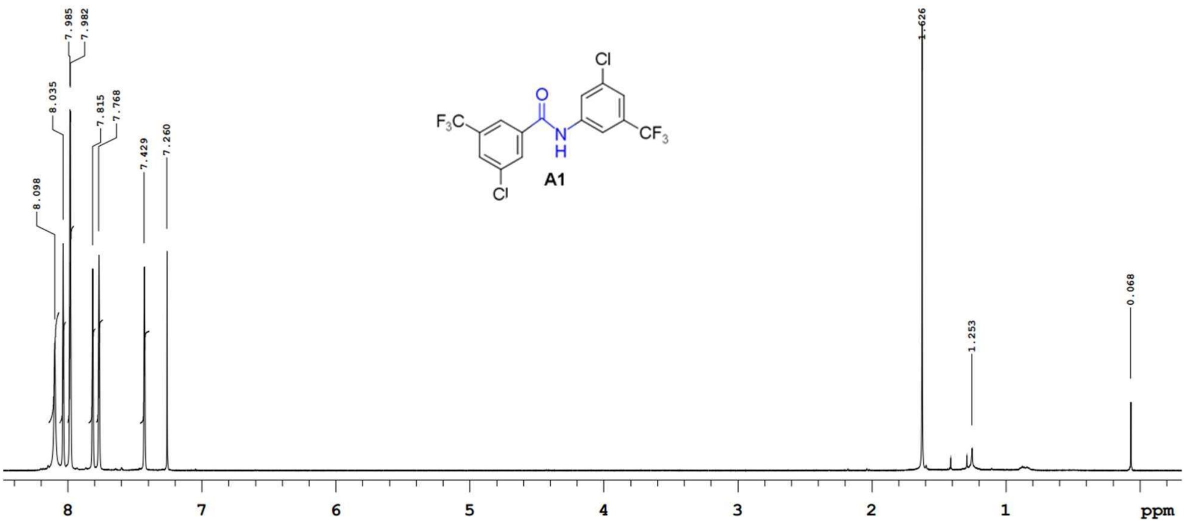

Figure S13. The 500 MHz ^{1}H NMR spectrum of A1.

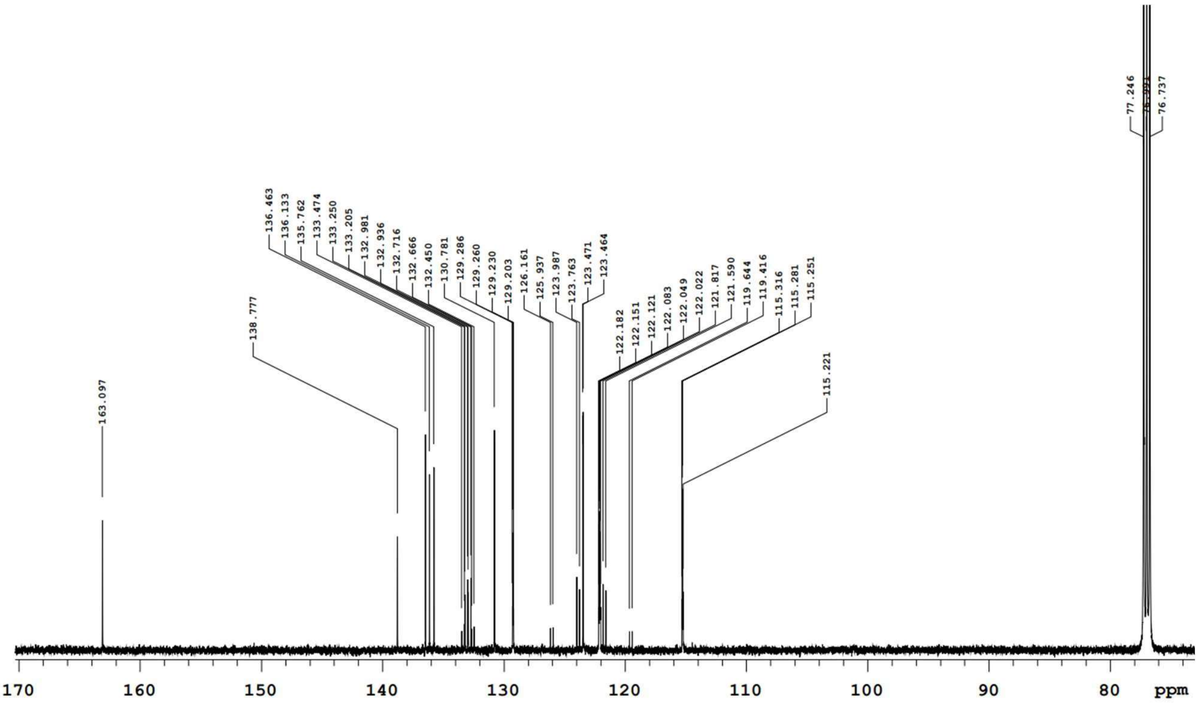

Figure S14. The 125 MHz ^{13}C NMR spectrum of A1.

4-(trifluoromethyl)-N-[4-(trifluoromethyl)phenyl]benzamide (A2).

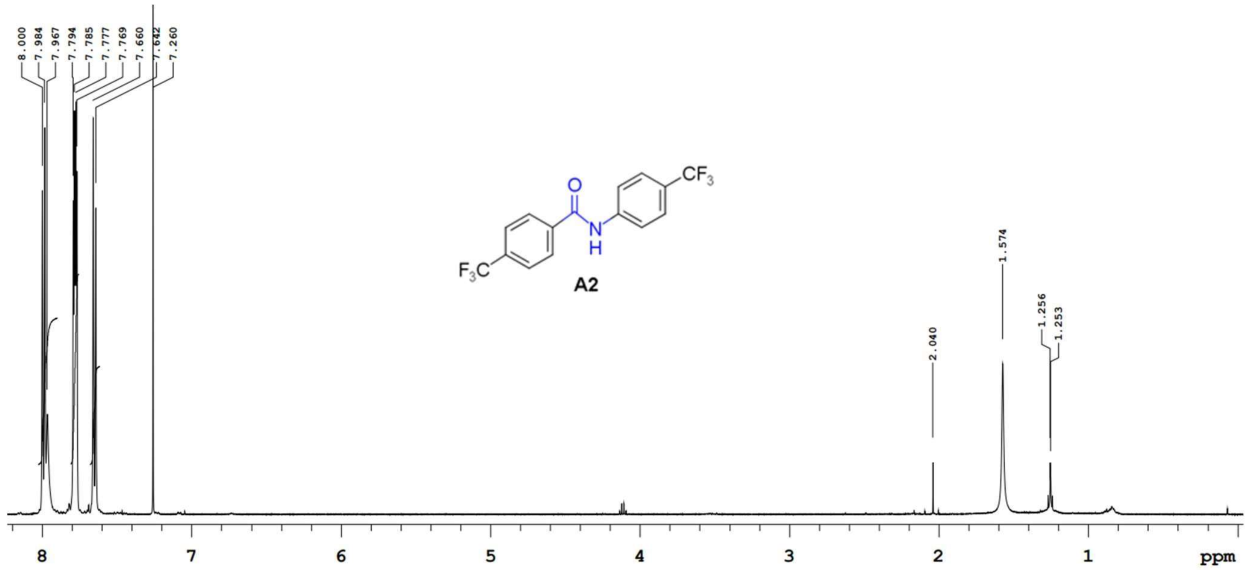

Figure S15. The 500 MHz ^1H NMR spectrum of A2.

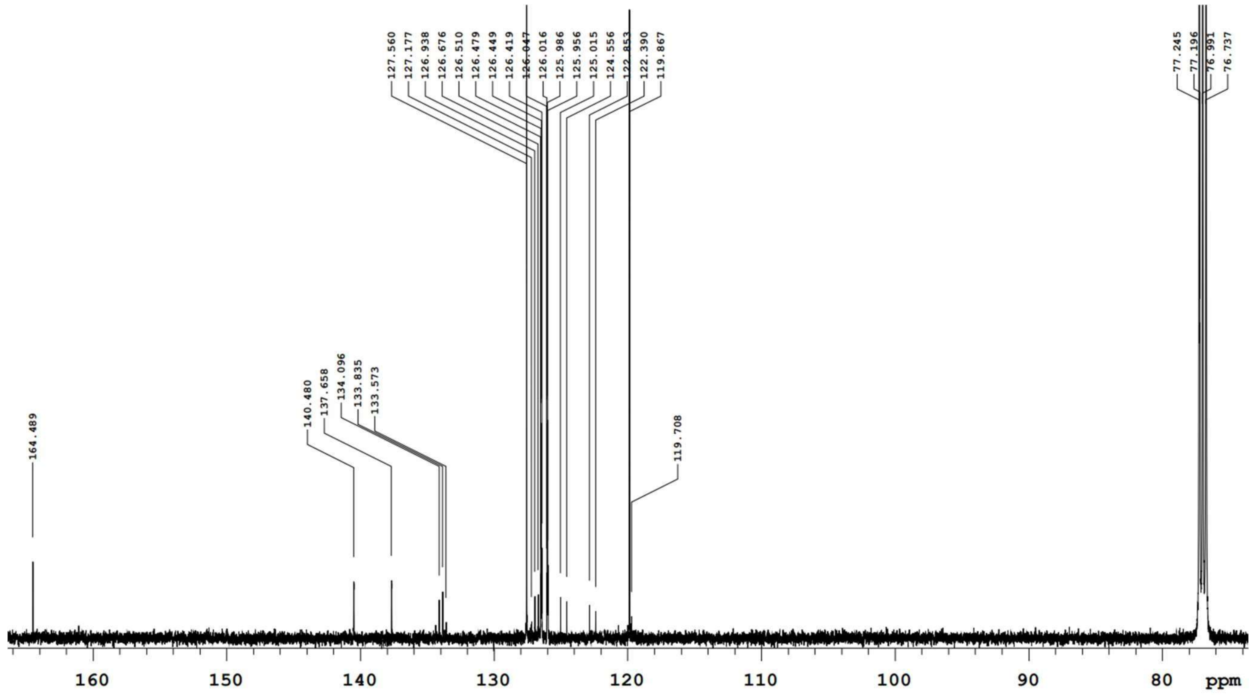

Figure S16. The 125 MHz ^{13}C NMR spectrum of A2.

4-(trifluoromethoxy)-N-[4-(trifluoromethoxy)phenyl]benzamide (A3).

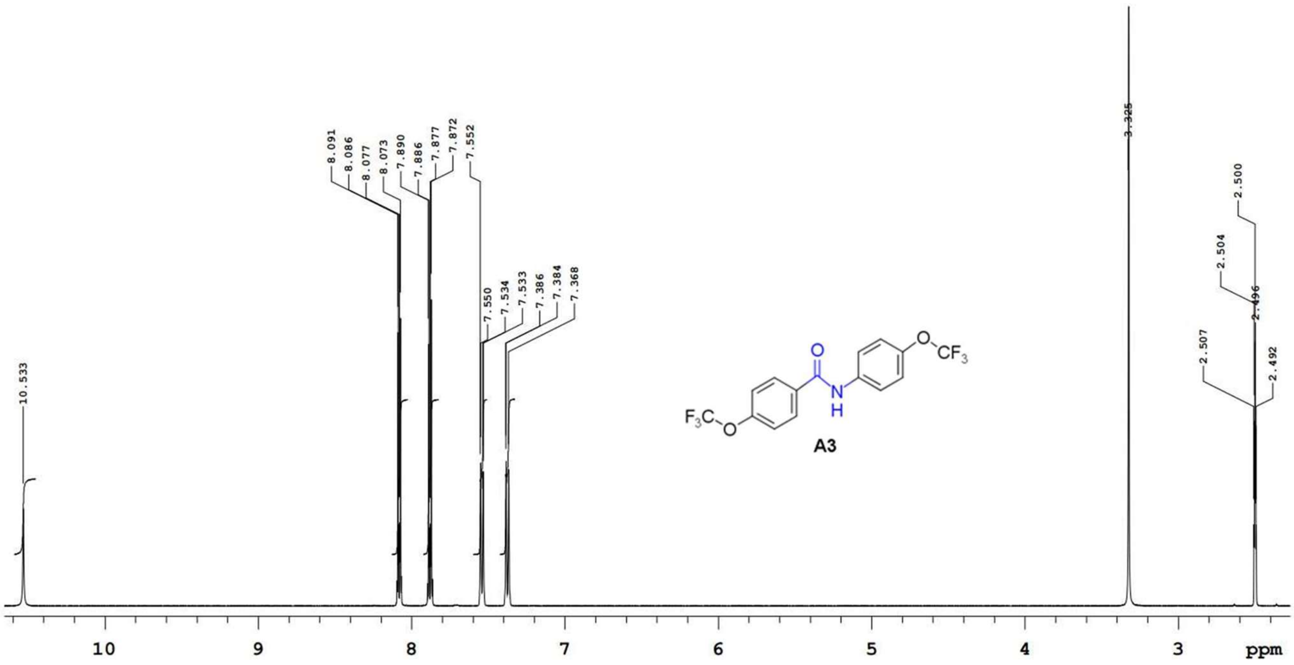

Figure S17. The 500 MHz ^{1}H NMR spectrum of A3.

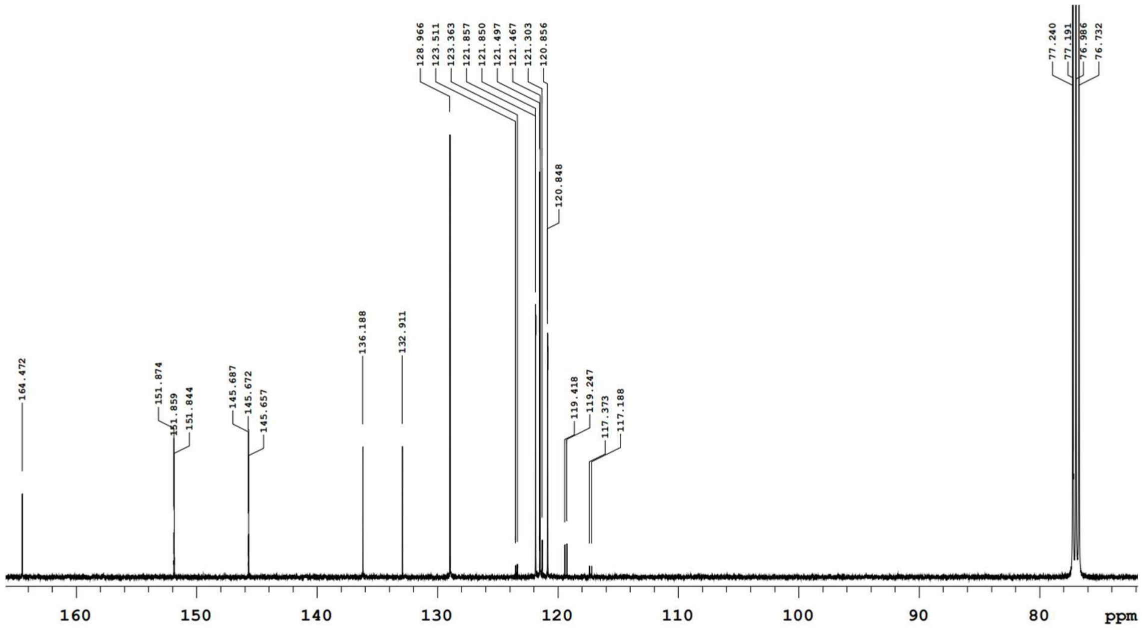

Figure S18. The 125 MHz ^{13}C NMR spectrum of A3 .

N-phenylbenzamide (A4).

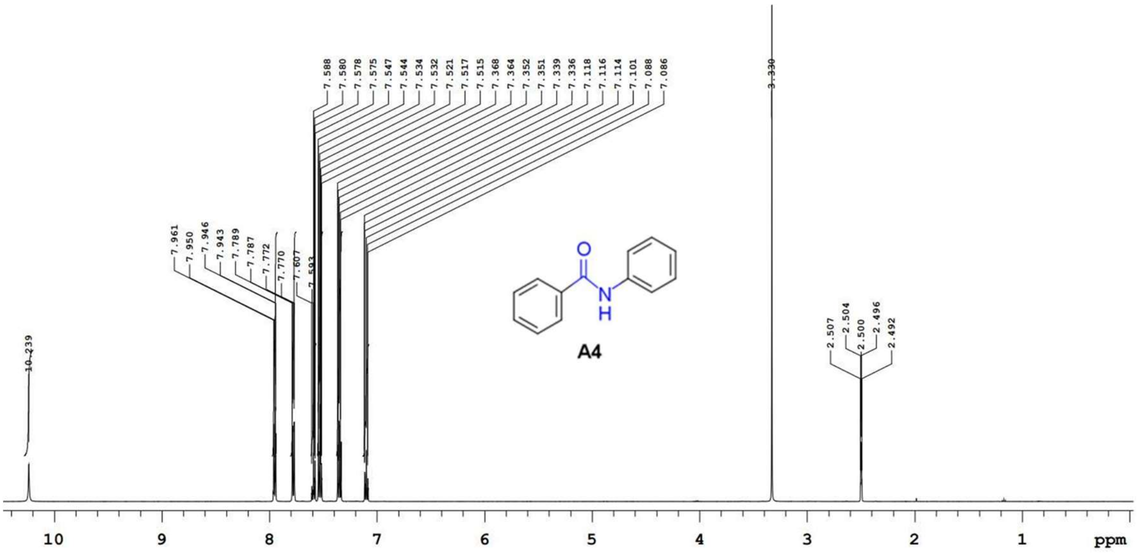

Figure S19. The 500 MHz ^{1}H NMR spectrum of A4.

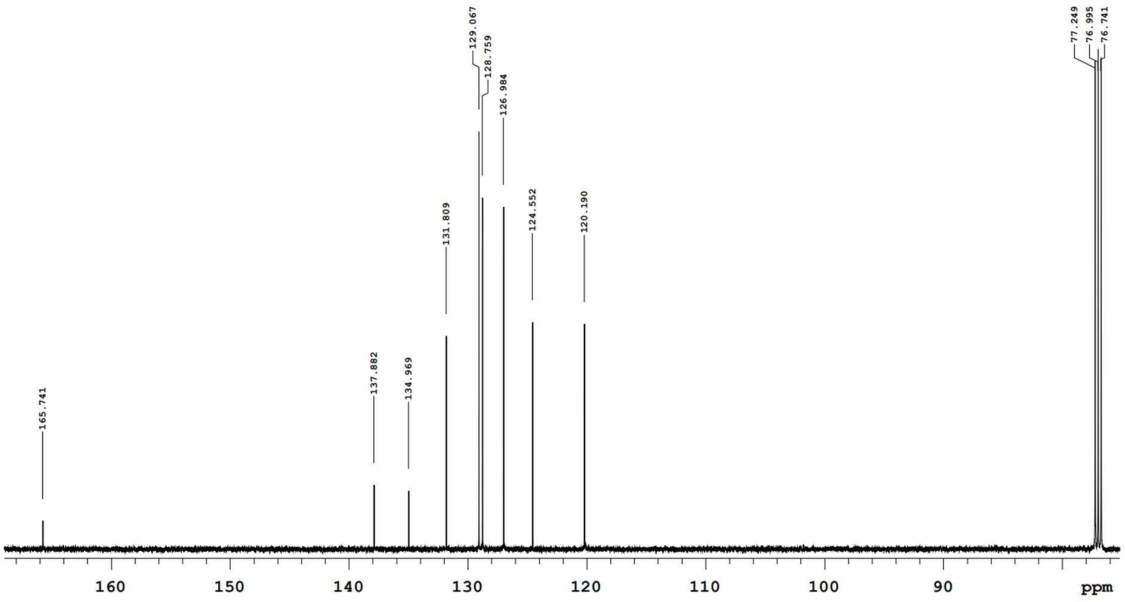

Figure S20. The 125 MHz ^{13}C NMR spectrum of A4.

3-[3-chloro-5-(trifluoromethyl)phenyl]-1-[6-({[3-chloro-5-(trifluoromethyl)phenyl]carbamoyl}amino)hexyl]urea (D1).

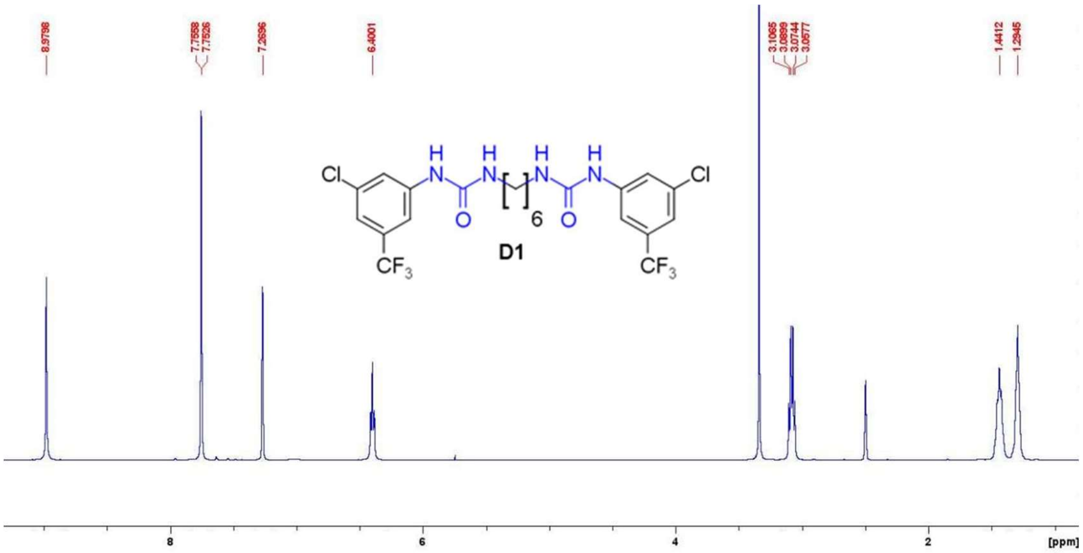

Figure S21. The 400 MHz ^{1}H NMR spectrum of D1.

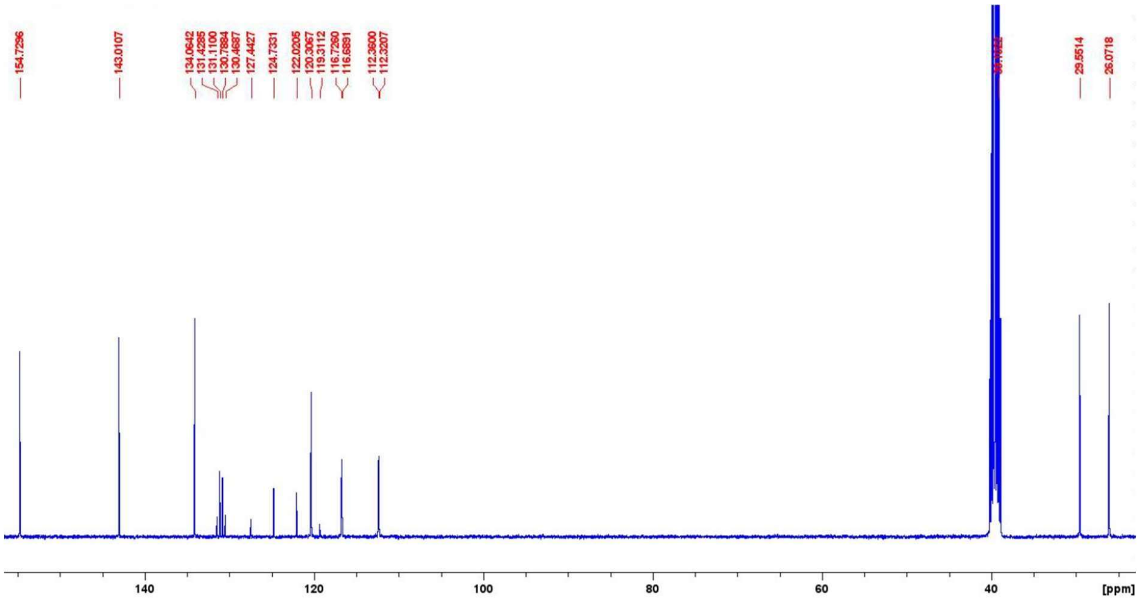

Figure S22. The 100 MHz ^{13}C NMR spectrum of D1.

3-[4-(trifluoromethyl)phenyl]-1-[6-({[4-(trifluoromethyl)phenyl]carbamoyl}amino)hexyl]urea (D2).

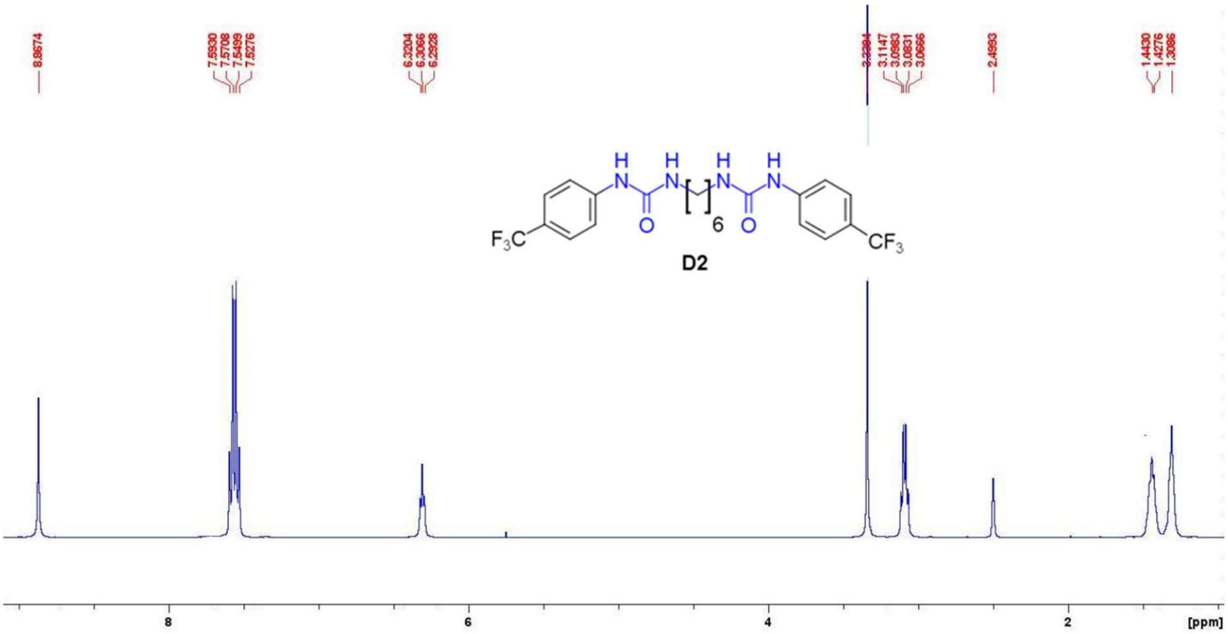

Figure S23. The 400 MHz ^{1}H NMR spectrum of D2.

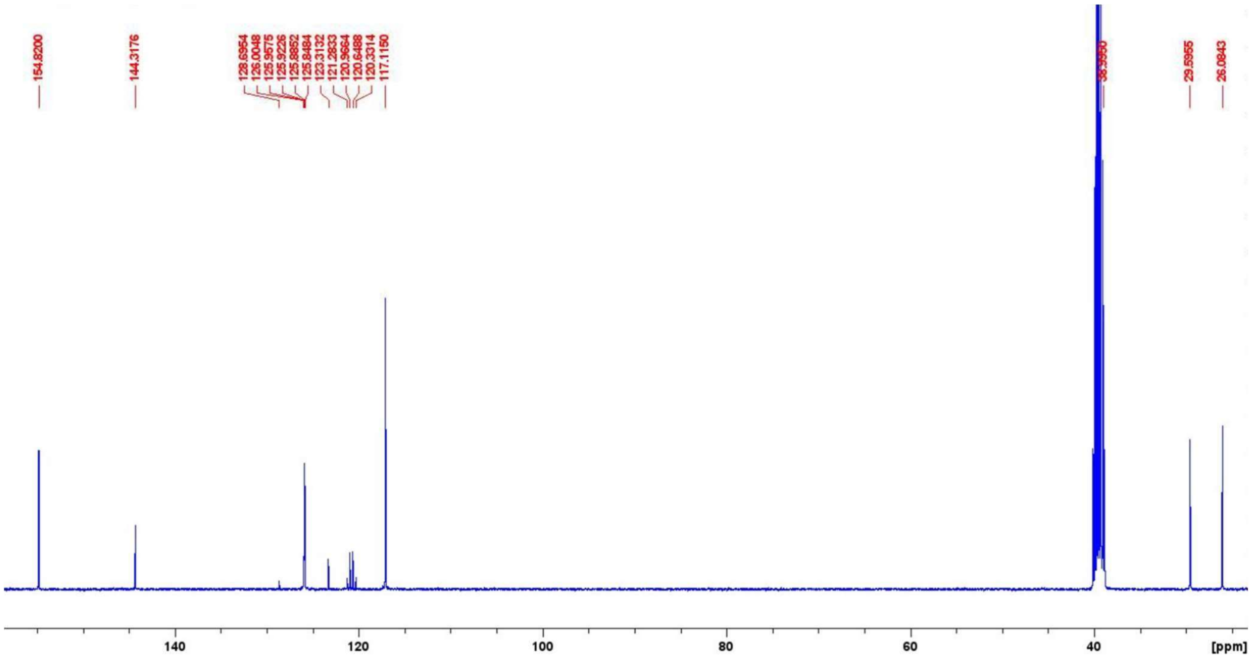

Figure S24. The 100 MHz ^{13}C NMR spectrum of D2.

3-[4-(trifluoromethoxy)phenyl]-1-[6-({[4-(trifluoromethoxy)phenyl]carbamoyl}amino)hexyl]urea (D3).

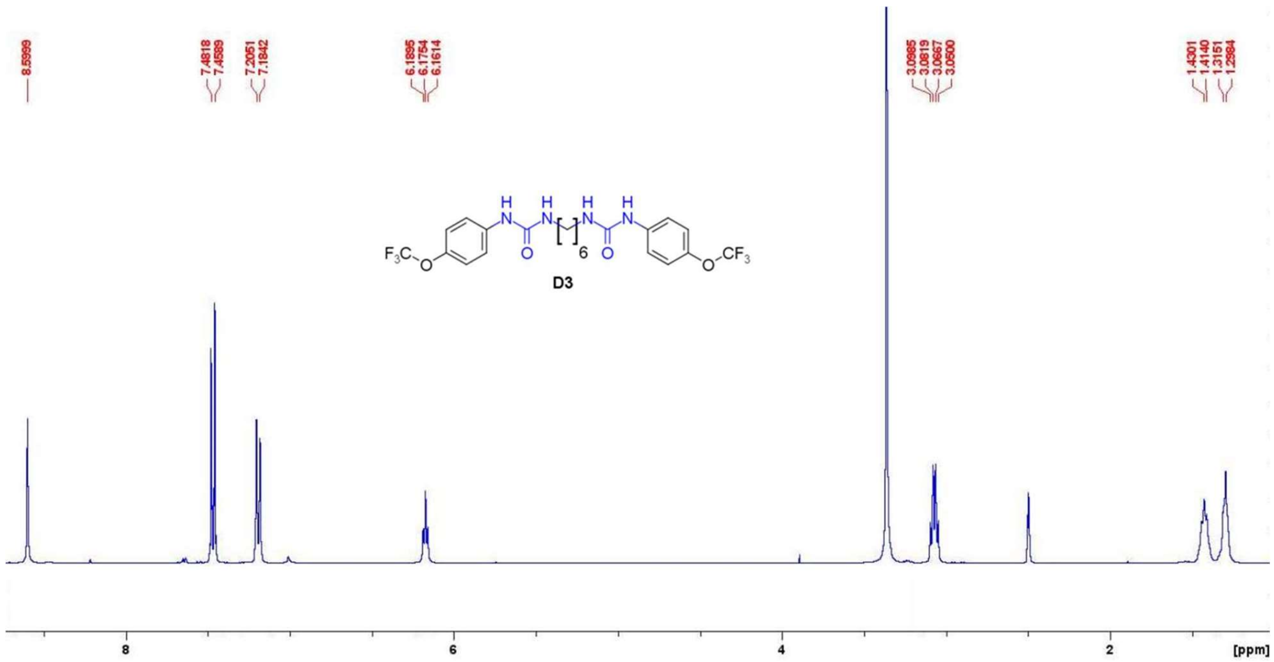

Figure S25. The 400 MHz ¹H NMR spectrum of D3.

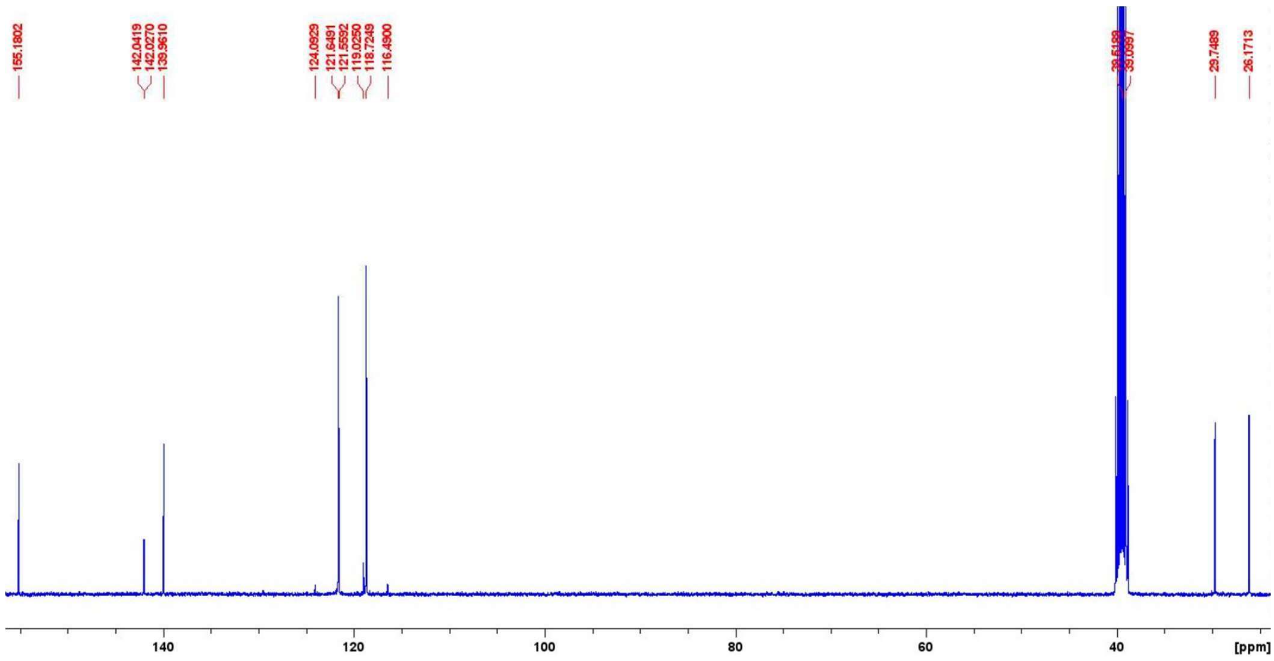

Figure S26. The 100 MHz ¹³C NMR spectrum of D3.

3-phenyl-1-{6-[(phenylcarbamoyl)amino]hexyl}urea (D4).

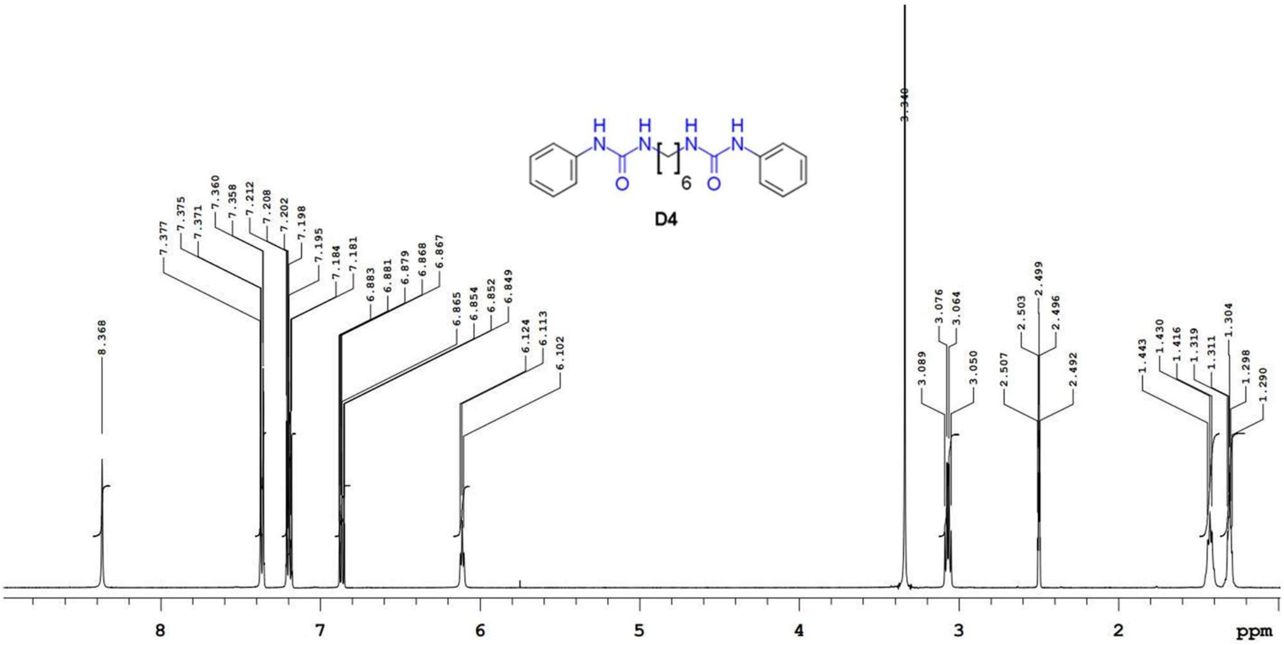

Figure S27. The 500 MHz ^{1}H NMR spectrum of D4.

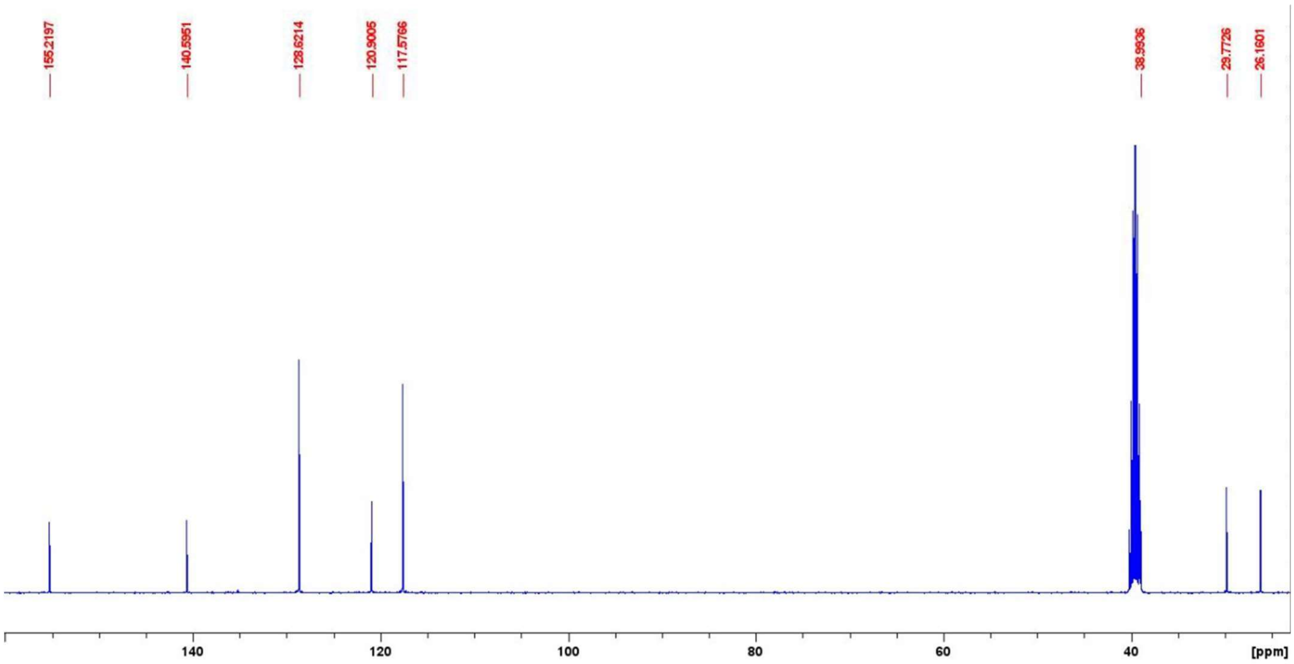

Figure S28. The 100 MHz ^{13}C NMR spectrum of D4.

1.8. S1/S4, A1/A4, D1/D4 HPTS Dose–Response Hill Analyses

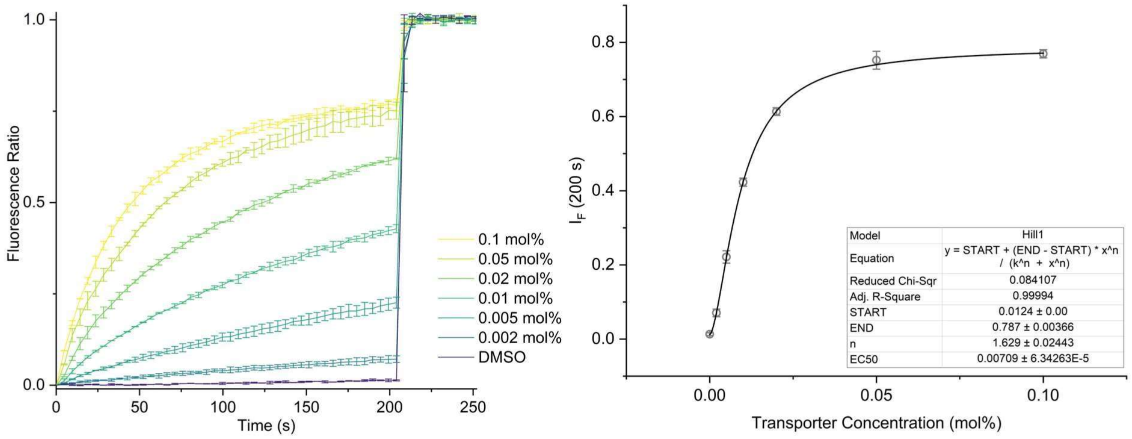

Figure S29. Hill plot analysis of H^+/OH^- transport facilitated by compound S1 measured using the KGluc assay. NaOH (5 mM) and valinomycin (0.05 mol%) were added to the vesicles before the addition of S1 at 0 s. Detergent was added at 205 s to lyse the vesicles. Compound concentrations are shown as compound-to-lipid molar ratios. Error bars represent standard deviations from at least two repeats.

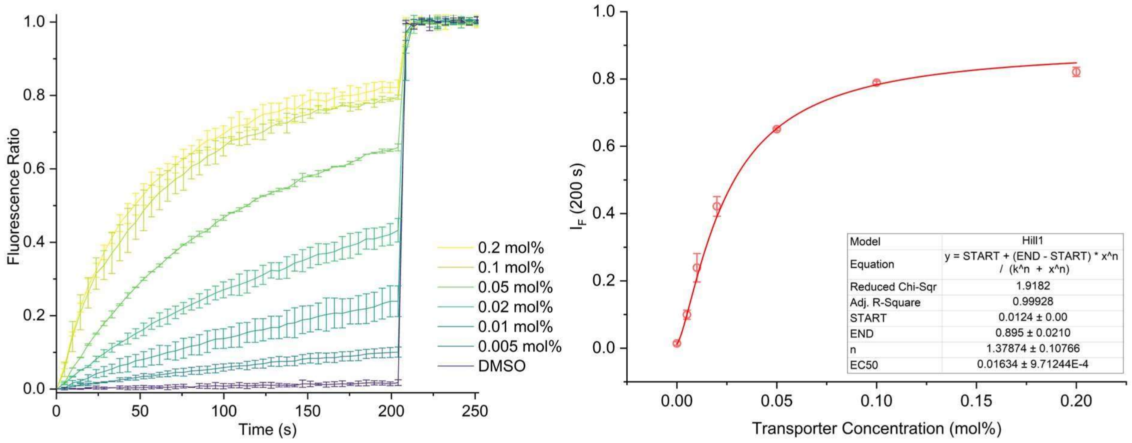

Figure S30. Hill plot analysis of H^+/OH^- transport facilitated by compound S1 measured using the KGluc assay. The vesicles were pre-treated with BSA (1 mol%). NaOH (5 mM) and valinomycin (0.05 mol%) were added to the vesicles before the addition of S1 at 0 s. Detergent was added at 205 s to lyse the vesicles. Compound concentrations are shown as compound-to-lipid molar ratios. Error bars represent standard deviations from at least two repeats.

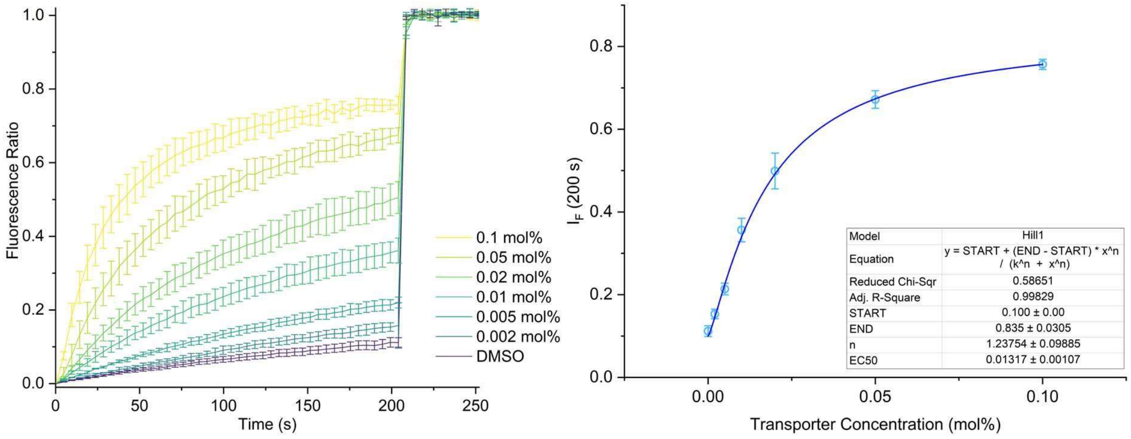

Figure S31. Hill plot analysis of H^+/OH^- transport facilitated by compound S1 measured using the KGluc assay. The vesicles were pre-treated with BSA (1 mol%). OA (10 mol%), NaOH (5 mM) and valinomycin (0.05 mol%) were added to the vesicles before the addition of S1 at 0 s. Detergent was added at 205 s to lyse the vesicles. Compound concentrations are shown as compound-to-lipid molar ratios. Error bars represent standard deviations from at least two repeats.

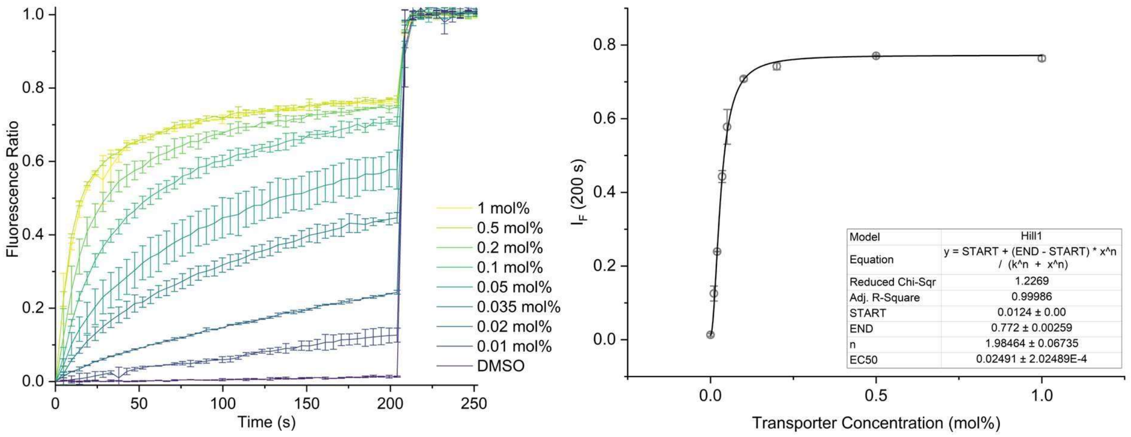

Figure S32. Hill plot analysis of H^+/OH^- transport facilitated by compound A1 measured using the KGluc assay. NaOH (5 mM) and valinomycin (0.05 mol%) were added to the vesicles before the addition of A1 at 0 s. Detergent was added at 205 s to lyse the vesicles. Compound concentrations are shown as compound-to-lipid molar ratios. Error bars represent standard deviations from at least two repeats.

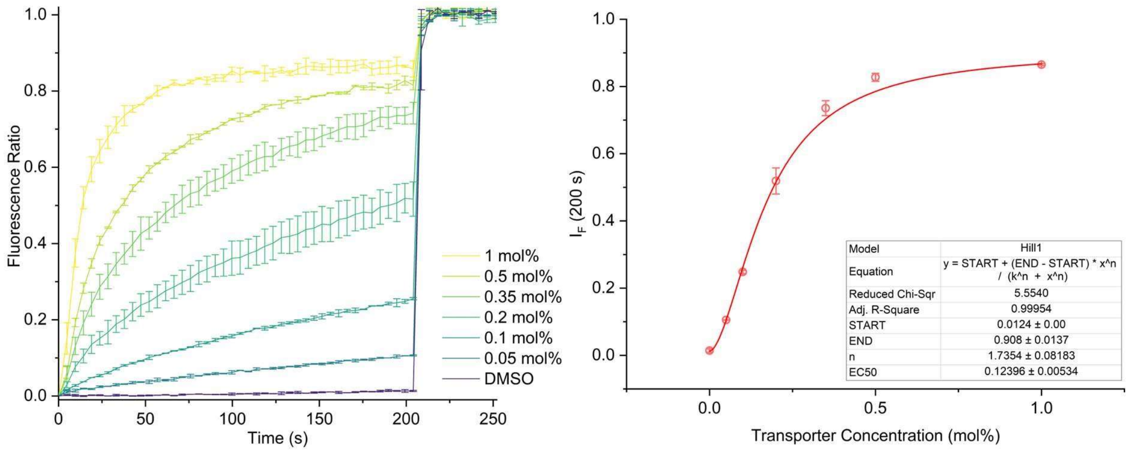

Figure S33. Hill plot analysis of H^+/OH^- transport facilitated by compound A1 measured using the KGluc assay. The vesicles were pre-treated with BSA (1 mol%). NaOH (5 mM) and valinomycin (0.05 mol%) were added to the vesicles before the addition of A1 at 0 s. Detergent was added at 205 s to lyse the vesicles. Compound concentrations are shown as compound-to-lipid molar ratios. Error bars represent standard deviations from at least two repeats.

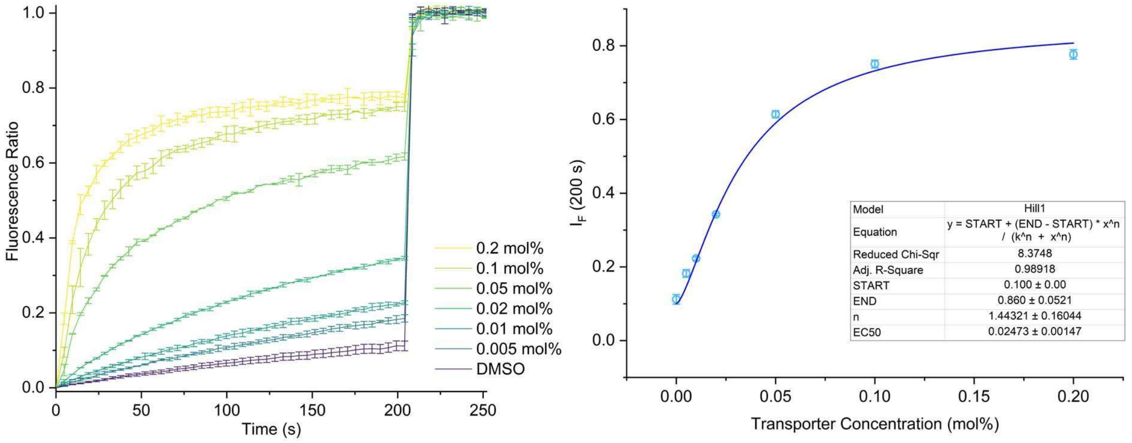

Figure S34. Hill plot analysis of H^+/OH^- transport facilitated by compound A1 measured using the KGluc assay. The vesicles were pre-treated with BSA (1 mol%). OA (10 mol%), NaOH (5 mM) and valinomycin (0.05 mol%) were added to the vesicles before the addition of A1 at 0 s. Detergent was added at 205 s to lyse the vesicles. Compound concentrations are shown as compound-to-lipid molar ratios. Error bars represent standard deviations from at least two repeats.

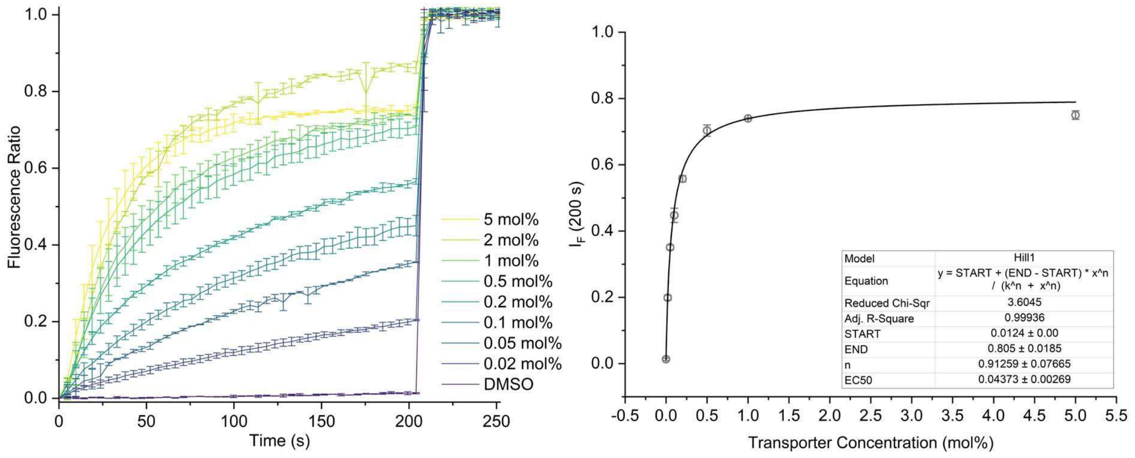

Figure S35. Hill plot analysis of H^+/OH^- transport facilitated by compound D1 measured using the KGluc assay. NaOH (5 mM) and valinomycin (0.05 mol%) were added to the vesicles before the addition of D1 at 0 s. Detergent was added at 205 s to lyse the vesicles. Compound concentrations are shown as compound-to-lipid molar ratios. Error bars represent standard deviations from at least two repeats.

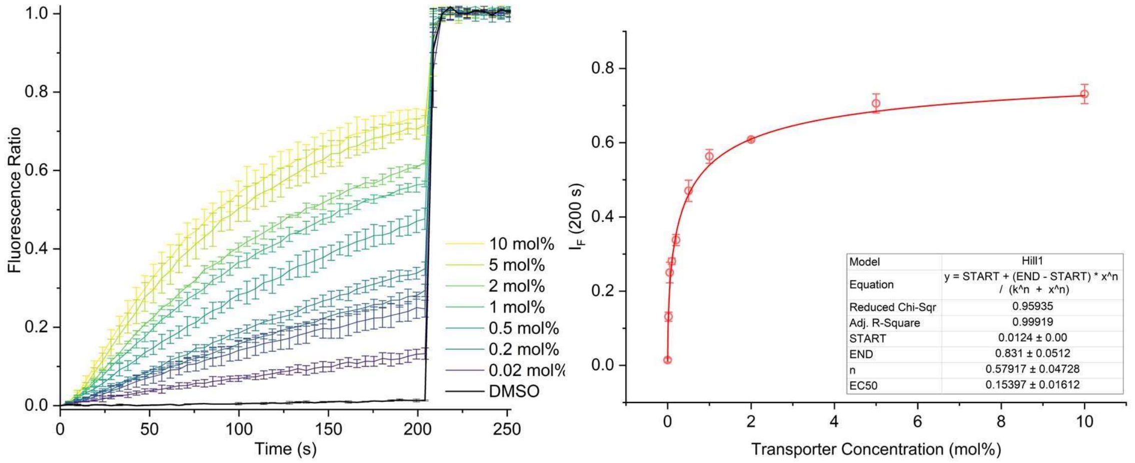

Figure S36. Hill plot analysis of H^+/OH^- transport facilitated by compound D1 measured using the KGluc assay. The vesicles were pre-treated with BSA (1 mol%). NaOH (5 mM) and valinomycin (0.05 mol%) were added to the vesicles before the addition of D1 at 0 s. Detergent was added at 205 s to lyse the vesicles. Compound concentrations are shown as compound-to-lipid molar ratios. Error bars represent standard deviations from at least two repeats.

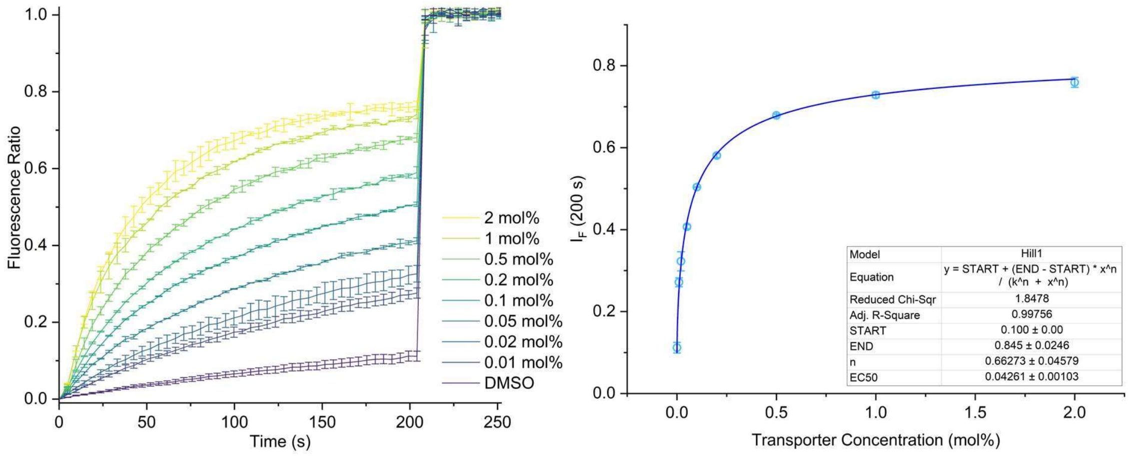

Figure S37. Hill plot analysis of H^+/OH^- transport facilitated by compound D1 measured using the KGluc assay. The vesicles were pre-treated with BSA (1 mol%). OA (10 mol%), NaOH (5 mM) and valinomycin (0.05 mol%) were added to the vesicles before the addition of D1 at 0 s. Detergent was added at 205 s to lyse the vesicles. Compound concentrations are shown as compound-to-lipid molar ratios. Error bars represent standard deviations from at least two repeats.

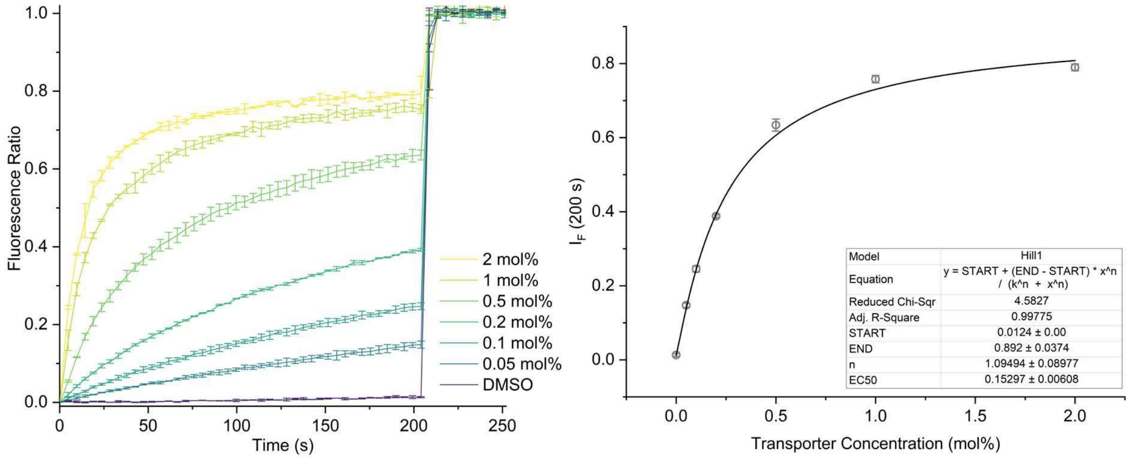

Figure S38. Hill plot analysis of H^+/OH^- transport facilitated by compound S4 measured using the KGluc assay. NaOH (5 mM) and valinomycin (0.05 mol%) were added to the vesicles before the addition of S4 at 0 s. Detergent was added at 205 s to lyse the vesicles. Compound concentrations are shown as compound-to-lipid molar ratios. Error bars represent standard deviations from at least two repeats.

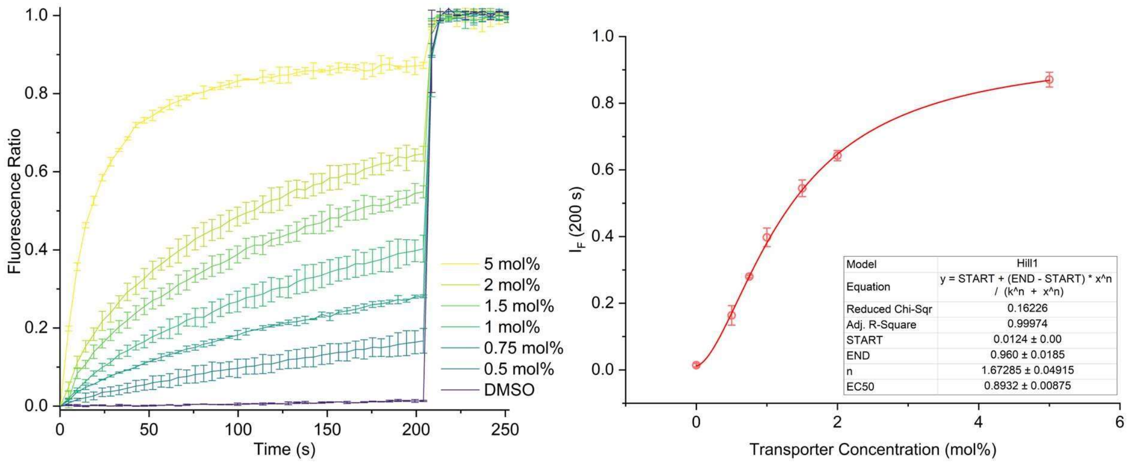

Figure S39. Hill plot analysis of H^+/OH^- transport facilitated by compound S4 measured using the KGluc assay. The vesicles were pre-treated with BSA (1 mol%). NaOH (5 mM) and valinomycin (0.05 mol%) were added to the vesicles before the addition of S4 at 0 s. Detergent was added at 205 s to lyse the vesicles. Compound concentrations are shown as compound-to-lipid molar ratios. Error bars represent standard deviations from at least two repeats.

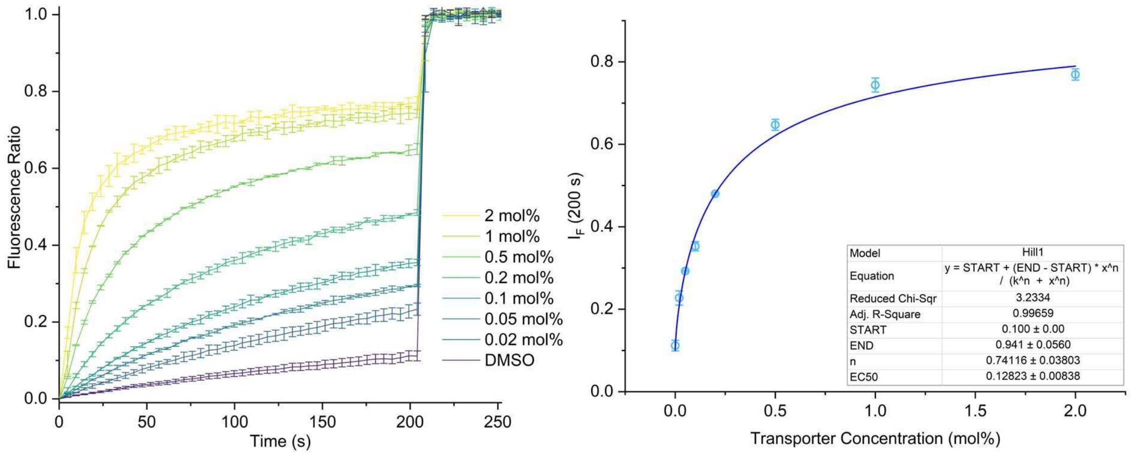

Figure S40. Hill plot analysis of H^+/OH^- transport facilitated by compound S4 measured using the KGluc assay. The vesicles were pre-treated with BSA (1 mol%). OA (10 mol%), NaOH (5 mM) and valinomycin (0.05 mol%) were added to the vesicles before the addition of S4 at 0 s. Detergent was added at 205 s to lyse the vesicles. Compound concentrations are shown as compound-to-lipid molar ratios. Error bars represent standard deviations from at least two repeats.

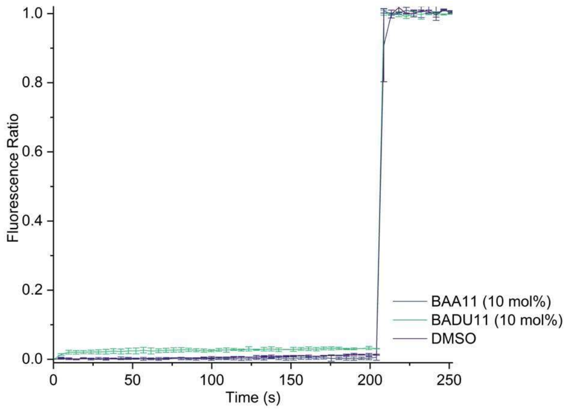

Figure S41. Efflux plots of H^+/OH^- transport facilitated by compound A4 and D4 measured using the KGluc assay. A 10 mol% compound-to-lipid molar ratio of either compound was added to the vesicles at 0 s and detergent was added at 205 s to lyse the vesicles. Error bars represent standard deviations from at least two repeats. H^+/OH^- efflux did not exceed 10% after 200 s under the conditions tested.

References

References in Supplementary Material follow main text numbering and are listed by order appearance below:

[27] Gilchrist, A.M.; Wang, P.; Carreira-Barral, I.; Alonso-Carrillo, D.; Wu, X.; Quesada, R.; Gale, P.A. Supramolecular methods: the 8-hydroxypyrene-1,3,6-trisulfonic acid (HPTS) transport assay. *Supramol. Chem.* **2021**, *33*, 325–344, https://doi.org/10.1080/10610278.2021.1999956.

[17] Cushman, M.; Georg, G.I.; Holzgrabe, U.; Wang, S. Absolute Quantitative ^{1}H NMR Spectroscopy for Compound Purity Determination. *J. Med. Chem.* **2014**, *57*, 9219–9219, https://doi.org/10.1021/jm501683w.

Disclaimer/Publisher's Note: The statements, opinions and data contained in all publications are solely those of the individual author(s) and contributor(s) and not of MDPI and/or the editor(s). MDPI and/or the editor(s) disclaim responsibility for any injury to people or property resulting from any ideas, methods, instructions or products referred to in the content.
